# An antibacterial, antioxidant and hemostatic hydrogel accelerates infectious wound healing

**DOI:** 10.1186/s12951-025-03148-w

**Published:** 2025-01-28

**Authors:** Ziyi Zhou, Dengjun Zhang, Xuchao Ning, Linbo Jin, Yijing Lin, Chen Liang, Xin Wen, Tianhao Huang, Junli Zhou, Yiming Zhang

**Affiliations:** 1 Department of Plastic and Cosmetic Surgery, Xinqiao Hospital, Army Medical University, Chongqing, 400037 China; 2 Department of Burn and Plastic surgery, The Tenth Affiliated Hospital of Southern Medical University (Dongguan People’s Hospital), Dongguan, China; 3 Department of Plastic Surgery, Cheeloo College of Medicine, Qilu Hospital of Shandong University (Qingdao), Qingdao, 266035 China

**Keywords:** Hydrogel, Antibacterial, Antioxidant, Hemostasis, Infected wound

## Abstract

Hydrogel drug-delivery system that can effectively load antibacterial drugs, realize the in-situ drug release in the microenvironment of wound infection to promote wound healing. In this study, a multifunctional hydrogel drug delivery system (HA@TA-Okra) was constructed through the integration of hyaluronic acid methacrylate (HAMA) matrix with tannic acid (TA) and okra extract. The composition and structural characteristics of HA@TA-Okra system and its unique advantages in the treatment of diverse wounds were systematically evaluated. TA, due to its unique chemical structure, is able to anchor within the HAMA network through interactions and cross-linking, conferring exceptional mechanical strength and stability to the hydrogel. Both TA and okra extract possess antioxidant and antibacterial properties, and when they two acts synergistically they can effectively scavenge free radicals, enhance antibacterial action, diminishing the risk of wound infection. In vitro experiments revealed that HA@TA-Okra system has superior properties, such as rapid gel response, remarkable swelling regulation, and potent antioxidant ability. Furthermore, the HA@TA-Okra system significantly outperformed conventional dressings in terms of hemostatic performance in a rat hemorrhage model. We further evaluated the repair role of HA@TA-Okra system in vivo by establishing an animal model of full-thickness skin defects and a model of infected total skin defects. The results confirmed its positive effects in fighting bacterial infection, reducing inflammation and promoting wound healing. In summary, the HA@TA-Okra system exhibits comprehensive properties such as antibacterial, antioxidant and hemostatic properties, which has a potential application in the field of tissue repair medicine.

**Graphical Abstract:**

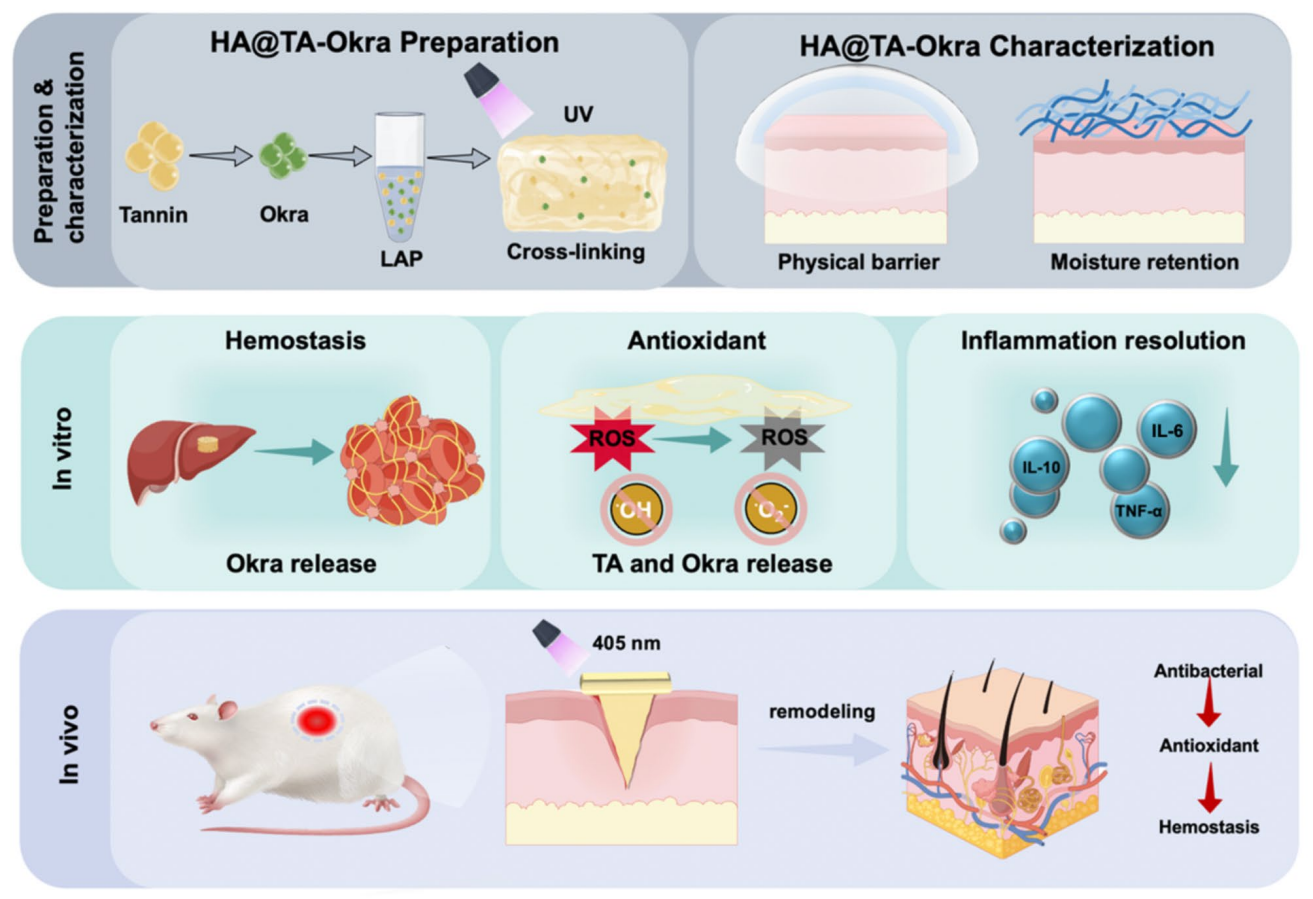

**Supplementary Information:**

The online version contains supplementary material available at 10.1186/s12951-025-03148-w.

## Introduction

The skin serves as the primary barrier against bacterial invasion from the external environment and protects our body from harm. When the skin is broken, untimely treatment or mismanagement can easily lead to bacterial infection, resulting in delayed wound healing and even tissue necrosis and sepsis [1–3]. Current studies have clarified that excessive oxidative stress is a pivotal factor that hinders tissue recovery during the healing process of infected wounds [4–6]. Abnormal accumulation of reactive oxygen species (ROS) in the wound area triggers a robust inflammatory cascade response. In addition, ROS can inhibit the activation and differentiation of endogenous stem cells and the immunomodulatory function of macrophages through intricate molecular mechanisms [7–9]. Therefore, regulating the balance between oxidative and antioxidant effects in the trauma microenvironment, and mitigating oxidative stress are important for promoting tissue recovery [10–12].

Hydrogel scaffolds have a wide range of applications in the biomedical field, especially in tissue repair and regeneration. Through precise design, the hydrogel scaffold can have a three-dimensional network structure with specific pores. This structure can mimic the microenvironment of natural tissues and provide a suitable living space for cells, which is conducive to cell adhesion, proliferation and differentiation. Meanwhile, hydrogel scaffolds are capable of loading and releasing bioactive molecules, such as antioxidants, thereby reducing cell and tissue damage caused by oxidative stress [13–19]. In addition, the degradation products of hydrogel scaffolds are usually non-toxic or low-toxic and can be absorbed or excreted by the body, which further reduces oxidative stress on tissues. Therefore, hydrogel scaffolds have significant advantages in reducing oxidative stress and promoting tissue recovery. In order to achieve good scavenging efficacy of reactive oxygen species, researchers have been exploring various hydrogel systems with smart response properties. These systems can dynamically adjust their ROS scavenging capacity in response to changes in the external environment (e.g., pH, temperature, specific molecule concentrations) [20–22]. The loading of antioxidants has become an effective strategy to realize the antioxidant function, with cerium oxide, conductive polymers, gallic acid, curcumin, among others, which have been successfully incorporated into hydrogel matrices [23–25].

TA stands out as a preferred candidate material for hydrogel integration due to its excellent antibacterial, antioxidant and anti-inflammatory properties [26–28]. In the acidic microenvironment of an infected wound, TA utilizes its unique polyphenolic structure to bind to proteins on bacterial cell walls, disrupting the microbial membrane structure and achieving an antibacterial effect. Under acidic conditions, TA can also be reduced to release potent free radical scavengers. The dual mechanism shows its powerful antibacterial efficacy. The incorporation of TA significantly enhances various functional attributes of the double-network hydrogels composed of poly (vinyl alcohol) (PVA) and poly (acrylic acid) (PAA) including toughness, self-healing, hemostasis, and antibacterial capacity [29]. Fe-modified molybdenum disulfide nanosheets (MoS_2_ @TA /Fe NSs) were chelated with TA and fixed on the hydrogel. The MoS_2_ @TA/Fe NSs endow the hydrogel with outstanding antioxidant capabilities, enabling it to scavenge excess ROS and reactive nitrogen species (RNS) under neutral conditions, thereby maintaining antioxidant system balance and preventing inflammation [30]. These studies show that TA can play its own advantages in the hydrogel system. Okra, as a natural resource rich in polysaccharides, vitamins, and minerals, has demonstrated significant potential in hemostasis and antioxidant activities through its extracts [31, 32]. Okra extract can activate coagulation factors in the blood, facilitating their rapid release, and enhancing platelet activation and adhesion, rapidly forming a hemostatic barrier at the wound site. Additionally, okra may further accelerate the healing process through its abundant nutrient content and bioactive substances. Therefore, okra extract has been favored by researchers for a long time and has become a hot spot in biomaterials research. Composite hydrogels possess their own unique functions, but it is still rare to be able to continue to play a role throughout the entire wound healing process.

Despite its ability to mimic the structure of the extracellular matrix and provide an environment for cell attachment and proliferation, the antimicrobial properties of conventional hydrogels are relatively limited, making it difficult to effectively contain wound infections. In addition, uncontrolled bleeding, another major challenge in wound management, may lead to serious consequences such as organ failure, increased morbidity, and even death. Ideal wound dressings should have excellent hemocompatibility and biocompatibility and be able to concentrate clotting factors as they absorb blood, resulting in rapid hemostasis. Therefore, it is important to develop innovative wound dressings to meet the urgent needs of modern medicine. These new dressings need to provide full coverage of wounds of all shapes and sizes, facilitate the natural healing process, provide strong antimicrobial protection, and be highly biocompatible for hemostasis [33].

Herein, we are employing HAMA, TA, and okra extract as key components, the HA@TA-Okra system was constructed through chemical reactions and physical crosslinking strategies. Specifically, the first crosslinking network was robustly formed based on hydrogen bonding interactions, while the second network was efficiently established via radical polymerization induced by ultraviolet light. This design endowed the hydrogel with superior injectability, with the core advantage of being able to fill and repair complex wounds or tissue defects. Through a series of systematic experiments, we have demonstrated that HA@TA-Okra hydrogel containing TA and okra extract plays a comprehensive role in the wound healing cascade. The HA@TA-Okra hydrogel not only possesses remarkable hemostatic and antibacterial properties, but also effectively eliminates reactive oxygen species in the wound area, mitigating inflammatory responses, and creating favorable conditions for healing of infected wounds. The development of the HA@TA-Okra hydrogel system has been a great boon to the field of infected wound repair.

## Materials and methods

### Materials and reagents

The HAMA hydrogel (molecular weight:150 kDa, degree of substitution: 30 ~ 40 DS%) and Leucine aminopeptidase (LAP) were procured from Engineering For Life (Suzhou, China). Tannic acid and lipopolysaccharide (LPS) were acquired from Sigma-Aldrich (USA). The Live/Dead Cell Staining Kit, DPPH assay kit and Reactive Oxygen Species staining kit were obtained from Solarbio (Beijing, China). The EdU-488 Cell Proliferation Assay Kit was sourced from Beyotime Biotechnology (Shanghai, China). Enzyme linked immunosorbent kits (ELISA) for Interleukin-(IL-6) (Cat.EM0004), Tumor Necrosis Factor-α (TNF-α) (Cat.EM0010), and Interleukin-10 (IL-10) (Cat.EM0001) were purchased from HUABIO (Hangzhou, China). The CCK-8 assay kit was acquired from GLPbio (Shanghai, China). Fetal bovine serum (FBS) was sourced from Gibco (USA). Penicillin-streptomycin and 0.25% trypsin were obtained from BioSharp (Beijing, China). Serum-free culture medium, 4,6-diamidino-2-phenylindole staining (DAPI), phosphate-buffered saline (PBS) and paraformaldehyde were procured from Solarbio (Beijing, China). Mouse fibroblast cells (L929) and mouse monocyte-macrophage leukemia cells (RAW264.7) were kindly provided by the Department of Plastic Surgery, Xinqiao Hospital, Army Medical University.

### Preparation and characterization of the different hydrogels

In this study, HAMA was initially dissolved in LAP to a final concentration of 4 w/v%, serving as the precursor solution for hydrogel formation. Subsequently, TA was separately dissolved and uniformly dispersed into the 4 w/v% HAMA hydrogel solution at predetermined mass ratios (1.25, 2.5, 5 w/v%), yielding composites designated as HA@TA_1.25_, HA@TA_2.5_, and HA@TA_5_. On this basis, okra was further incorporated into 4 w/v% HAMA hydrogel containing 2.5 w/v% TA in different proportions. Finally, HA@TA-Okra_0.25_, HA@TA-Okra_0.5_, HA@TA-Okra_1_, and HA@TA-Okra_2_ series composites with okra concentrations of 0.25, 0.5, 1 and 2 w/v% were obtained. These composites were successfully fabricated into different kinds of hydrogels using UV-crosslinking technology (405 nm).

The injectability of hydrogels was evaluated following the methodology reported in previous literature [34]. Specifically, the hydrogel solution was placed in a syringe and extruded through a standard needle, and its injection fluency and morphology retention capabilities were directly observed and recorded. To validate the gelation efficacy, the hydrogel solutions were placed in transparent glass vials. After performing UV crosslinking for 30 s, 405 nm, the gelation ability was observed. The microstructures of the hydrogels were examined using scanning electron microscope (SEM, Phenom Pure, Holland). Fourier transform infrared spectroscopy (FTIR, Nicolet iS50, USA) was employed to analyze the chemical composition of the hydrogels. The hydrophilicity of the hydrogels was assessed using a contact angle meter (OCA20, DataPhysics, Germany). To quantify their swelling capacity, hydrogels of initial mass (*W*_*0*_) were immersed in PBS at 37 °C for 24 h. After removing excess surface moisture and weighing to obtain the wet mass (*W*_*w*_), the swelling ratio was calculated according to the formula: Swelling ratio (%) = (*W*_*w*_-*W*_*0*_)/*W*_*0*_ × 100%.

Following the protocol established in prior studies [1], the degradation properties of the hydrogel were evaluated. Hydrogel of initial mass (*W*_*0*_) were submerged in PBS at 37 °C and agitated continuously in a shaker at 120 rpm/min. Samples were retrieved at predetermined time points, freeze-dried, and their residual mass (*W*_*t*_) recorded. The degradation rate was then computed using the formula: Degradation (%) = *W*_*0*_- *W*_*t*_/*W*_*0*_ × 100%, providing a comprehensive analysis of the composite scaffolds’ stability under physiological conditions.

### Determination of TA and okra extracts concentrations and their effects on cytotoxicity and antioxidant properties

Four types of hydrogels—HAMA, HA@TA_1.25_, HA@TA_2.5_, and HA@TA_5_—were immersed in complete culture medium at a ratio of 0.1 g/mL for 24 h, collecting hydrogel extracts. Subsequently, the CCK-8 assay was employed to assess the cytotoxicity of these hydrogel. L929 cells were seeded in 24-well plates at a density of 1 × 10^4^ cells per well and cultured by hydrogel extracts under conditions of 37 °C and 5% CO_2_. At the same time points on days 1, 3 and 5, fresh complete medium containing 10%CCK-8 reagent was added to each well. Then the hydrogels were incubated in an incubator at 37℃, and then the absorbance was measured at 450 nm using a microplate reader (Thermo, US) to evaluate the compatibility. The antioxidant properties of the hydrogels were determined through their ability to scavenge 2,2-diphenyl-1-picrylhydrazyl (DPPH) free radicals, as previously documented [35]. Adhering to the DPPH assay protocol, vitamin C served as the positive control, and the absorbance was measured at 515 nm microplate reader. The antioxidant activity was calculated as per the manufacturer’s instructions and expressed as a percentage.

In a sterile environment, 500 µL of each of the four types of hydrogels-HAMA, HA@TA_1.25_, HA@TA_2.5_, and HA@TA_5_—were added to 24-well plates. An untreated hydrogel served as the control group. *Escherichia coli* (*E. coli*) and *Staphylococcus aureus* (*S. aureus*), at a concentration of 1 × 10^5^ CFU/mL (100 µL), were inoculated onto the surface of the hydrogel samples during their logarithmic growth phase and incubated at 37 °C for 4 h. Following incubation, the bacteria were washed with sterile PBS, and 100 µL of the wash solution was plated onto agar plates. After a further 24-hour incubation at 37 °C, bacterial growth was monitored and assessed. After extracting the leachates from the samples of HAMA, HA@TA-Okra_0.25_, HA@TA-Okra_0.5_, HA@TA-Okra_1_, and HA@TA-Okra_2_, an appropriate okra concentration was identified using the CCK-8 assay method mentioned above.

### Assessment of biocompatibility, migration, and antibacterial capacity of HA@TA-Okra_0.5_ hydrogels

In order to further explore the performance of HA@TA-Okra_0.5_, HAMA, the HA@TA_2.5_, and HA@Okra_0.5_ (HA@Okra for short)were set as the experimental control group. The overall cellular proliferation was quantitatively assessed employing the BeyoClick™ EdU Cell Proliferation Kit with Alexa Fluor 488. Briefly, a suitable number of cells were cultured in 24-well plates and subjected to various hydrogel extract stimulations. Following EdU labeling, the cells were stained according to the kit’s protocol, and observations and photographs were taken under an inverted microscope (Olympus, Japan).

To investigate the growth and migration of cells post-hydrogel treatment, a cell migration assay was performed. Specifically, L929 cells were seeded at a density of 1 × 10^5^ cells/well in 12-well plates. After monolayer formation, these cells were subjected to an overnight starvation treatment. A sterile pipette tip was used to create a scratch perpendicular to the plane of the fused monolayer. After washing off cell debris with sterile PBS, the cells were incubated with the different hydrogel extracts in a 5% CO_2_ atmosphere at 37 °C. At 0 h (*A*_*0*_), 12 h (*At*), and 24 h (*At*), cells were stained for 15 min at 37 °C using calcein from the live/dead staining kit, and images were captured under an inverted microscope. The degree of scratch closure was quantitatively evaluated using ImageJ software. The cell healing rate was calculated using the following formula: Wound healing (%) = *A*_*t*_ /*A*_*0*_ × 100%.

In a sterile environment, hydrogels of HAMA, HA@TA, HA@Okra, and HA@TA-Okra were prepared by dispensing 500 µL of each sample into 24-well plates, with untreated samples serving as the control group. Refer to Sect. 2.3 for specific antibacterial procedures. Bacterial colonies on the agar plates were photographed and analyzed using ImageJ software. Additionally, the samples were immersed in 10 mL of bacterial suspension and incubated with shaking for 24 h. Subsequently, the bacteria were stained using a live/dead staining kit, and their viability was observed under an inverted fluorescence microscope to further assess the antibacterial efficacy of the diverse hydrogels.

### Antioxidant activity and detection of inflammatory cytokine secretion

The DPPH radical scavenging capability of the HA@TA-Okra hydrogel was determined using a method consistent with Sect. 2.3 of this study. To investigate the intracellular ROS levels in RAW264.7 cells under LPS stimulation, the DCFH-DA labeling method was employed. RAW264.7 cells were seeded into 24-well plates at a density of 2 × 10^4^ cells/mL and cultured for 24 h. The cells were then stimulated with 1 µg/mL LPS for 24 h, with or without the addition of hydrogel extracts. Following stimulation, the cells were stained with DCFH-DA (10 µM) in the dark for 30 min. Intracellular ROS levels were quantified by measuring the fluorescence intensity under an inverted fluorescence microscope.

To gain insights into the modulatory effects of HA@TA-Okra hydrogels on cellular inflammatory responses, the concentrations of key inflammatory cytokines, including TNF-α, IL-6, and IL-10, were quantified using the ELISA method. Briefly, RAW264.7 cells were seeded into 24-well plates at a density of 1 × 10^5^ cells/well and co-incubated with hydrogel extracts for 24 h. After incubation, the supernatants were collected and centrifuged to remove dead cells and cellular debris. Subsequently, the concentrations of TNF-α, IL-6, and IL-10 were precisely determined using ELISA kits. By comparing the differences in cytokine concentrations among various treatment groups, the impact of hydrogels on cellular inflammatory responses and their potential immunomodulatory mechanisms could be objectively evaluated.

### In Vitro evaluation of hemolysis, blood coagulation, and platelet adhesion on HA@TA-Okra hydrogels

To assess the blood compatibility of HA@TA-Okra hydrogels, fresh sheep blood was utilized, adhering to the protocols established in previous studies [36]. Specifically, a 5% volume fraction of erythrocyte suspension was prepared from the sheep blood. Subsequently, 400 µL of hydrogels from distinct samples were immersed in 10 mL of PBS for 48 h. The resulting hydrogel extracts were then mixed with an equal volume of the 5% erythrocyte suspension and incubated for 1 h. Following centrifugation at 2000 rpm for 5 min, the absorbance of the supernatant was measured at 540 nm. As part of the assay, the supernatant from 1 mL of the erythrocyte suspension (*A*_*t*_) was centrifuged, and 1 mL of PBS was added as a negative control (*A*_*n*_), while 1 mL of deionized water served as the positive control (*A*_*p*_). The hemolysis ratio was calculated using the formula: Hemolysis Ratio (%) = (*A*_*t*_ - *A*_*n*_) / (*A*_*p*_ - *A*_*n*_) × 100%.

The coagulation assay was conducted in accordance with protocols reported in the literature. Precisely weighed samples and gauze (35 mg) were added to 100 µL of whole blood, which was then incubated at 37 °C for 10 min. Following this, 10 µL of 0.2 M CaCl_2_ solution was introduced, along with 10 mL of PBS, and the blood diffusion was observed. The absorbance of the supernatant at 540 nm (*A*_*s*_) was measured using a microplate reader. As a negative control (*A*_*0*_), deionized water was mixed with 100 µL of whole blood and 10 µL of 0.2 M CaCl_2_ solution. The blood clotting index (BCI) was determined using the equation: BCI (%) = *A*_*s*_ / *A*_*0*_ × 100%.

For the evaluation of platelet adhesion, 100 µL of whole blood was dripped onto the surface of the samples, which were then incubated at 37 °C for 1 h. Subsequently, the physically adhered blood was removed through rinsing with PBS. The samples were then fixed through a graded dehydration process using 4% paraformaldehyde and ethanol, followed by observation under a SEM.

### In vivo hemostatic performance

The hemostatic capabilities of the hydrogel scaffolds on non-compressible wounds were evaluated in SD rats (200–250 g, male) using tail amputation and liver incision models. This study was approved by the Institutional Animal Care and Use Committee of the Army Medical University. All animal housing and experimental procedures adhered to the guidelines set forth by the National Research Council for the Care and Use of Laboratory Animals. The hemostatic ability of HAMA and HA@TA-Okra hydrogels were investigated, with untreated samples and gauze serving as the control groups. Circular hydrogels with a diameter of 1 cm were prepared to fit the wound sites. Rats were anesthetized with sodium pentobarbital, and their skin was incised to expose the liver. A 1 cm incision was made on the surface of the liver and the hydrogel samples were applied to the bleeding site. In the tail amputation model, one-third of the rat’s tail was amputated, and the wound was covered with the samples under gentle pressure. The blood loss was recorded (*n* = 4) after confirming the absence of bleeding at the injury site.

### In vivo wound healing treatment and evaluation

Two animal models were established for wound healing experiments: a full-thickness defect model and an infected full-thickness skin defect model, utilizing SD rats (200–250 g, male). The animal experiments were conducted with the approval of the Animal Ethics Committee of the Army Medical University (Ethics No. AMUWEC20235104). Briefly, SD rats were randomly divided into four groups (*n* = 4), including an untreated control, gauze, HAMA hydrogel, and HA@TA-Okra hydrogel. Rats were anesthetized with pentobarbital sodium and shaved on their backs. Skin wounds, approximately 15 mm in diameter and 2 mm in depth, were created using sterile scissors. For the infected full-thickness skin defect model, each wound was inoculated with *S. aureus* (100 µL, 10^6^ CFU mL^− 1^) for 1 h to ensure the establishment of infected wounds [37]. Each wound was coated with 200 µL hydrogel until it was lever with the wound bed. Wounds were photographed at 0, 3, 7, 10, and 14 days, and wound areas were accurately calculated using ImageJ software.

### Histological and immunohistochemical insights

To comprehensively understand the histological alterations and immune responses during wound healing, samples of wound tissues and major internal organs (heart, liver, spleen, lungs, and kidneys) were collected at days 3, 7 and 14. Following standard protocols, samples were fixed in 4% paraformaldehyde, embedded in paraffin, and sectioned. Histological observations were conducted using H&E staining and Masson’s trichrome staining to reveal the microstructure of wound healing and collagen fiber deposition. All sections were meticulously analyzed and recorded using an upright microscope (Nikon, Japan).

Furthermore, to explore the immunomodulatory mechanisms in regenerated skin, immunofluorescence staining analysis was performed. On day 14, excised regenerated skin tissues were used to detect the expression of key inflammatory cytokines, IL-10 and TNF-α. Through specific antibody and DAPI nuclear staining, the expression patterns of these cytokines were observed under an inverted fluorescence microscope.

### Statistical analysis

Comprehensive data analysis was conducted using GraphPad Prism 6 software to ensure the accuracy and reliability of results. All experiments were independently repeated at least three times to verify the stability and reproducibility of the findings. Experimental data are presented as mean ± standard deviation (SD), and the significance of intergroup differences was tested using one-way ANOVA. where all error bars are SD. Statistical significance levels were set at *P < 0.05, **P < 0.01, and ***P < 0.001 to clearly distinguish whether the differences in effects between different treatment groups were statistically meaningful.

## Result and discussion

### Characterization and performance analysis of different hydrogels

**Fig. 1:**
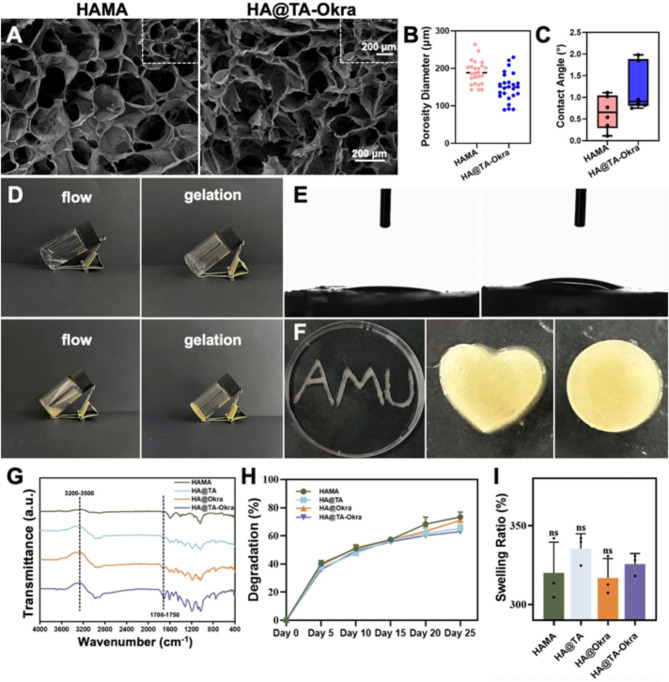
Characterization of HA@TA-Okra Hydrogels. (**A**) SEM image of hydrogels. (**B**) Pore size analysis of hydrogels. (**C**) Hydrogels contact angle box chart statistics. (**D**) HAMA and HA@TA-Okra Hydrogels formed by UV irradiation (405 nm, 30 s). (**E**) Contact angle of the hydrogels. (**F**) Injectability assessment of HA@TA-Okra Hydrogels. (**G**) FTIR spectra of HAMA, HA@TA, HA@Okra, and HA@TA-Okra. (**H**) Degradation ratio of hydrogels. (**I**) Swelling ratio of hydrogels

The extracellular matrix (ECM) is a three-dimensional scaffolding structure composed of proteins and polysaccharides secreted by cells. It not only provides physical support for cells, but also participates in the regulation of cell behavior and plays a crucial role in maintaining the normal structure and function of the skin [38, 39]. In this study, we designed a novel HAMA hydrogel that is capable of rapid light curing. It can mimic the structure of natural ECM to provide attachment sites for cells, which facilitates cell proliferation and differentiation. As depicted in Fig. 1A and B, the HAMA and HA@TA-Okra hydrogel exhibited a uniformly distributed porous structure with pore diameters measured to be approximately 188 ± 31 μm and 150 ± 37 μm, respectively. Notably, the addition of TA and okra components hardly changed the original pore structure of HA@TA-Okra hydrogel. As observed in Figure S1, the shape of the two hydrogels transformed into a three-dimensional structure after freeze-drying treatment. This three-dimensional structure not only maintains the original loose and porous properties of the hydrogel, but also further enhances its stability and application range. This property enables HAMA and HA@TA-Okra hydrogels not only to promote the absorption of wound exudate but also to efficiently transport nutrients, Further performance evaluation of the hydrogels revealed that both HAMA and HA@TA-Okra hydrogels exhibited remarkable hydrophilicity, with contact angle measurements of approximately 1° and 2°, respectively (Fig. 1C and E). This characteristic ensures efficient water absorption and retention, fostering a moist environment in the wound area, thereby accelerating the healing process.

Moreover, HAMA and HA@TA-Okra hydrogels all boast exceptional injectability, allowing precise delivery to the target site in a liquid state, followed by rapid solidification under UV light exposure to form a stable three-dimensional structure (Fig. 1D). This property can also meet the needs of wound repair of diverse shapes, as shown in Fig. 1F HA@TA-Okra hydrogel patterns of various shapes. This feature simplifies surgical procedures and enhances the precision and flexibility of treatment. As shown in Fig. 1G, a significant wide absorption band was observed in the wavenumber range of 3200–3500 cm^− 1^, which was attributed to the characteristic vibration caused by the hydroxyl (OH) groups rich in HAMA, TA and okra. Based on spectroscopic analysis, we inferred that a hydrogen bond interaction may be formed between HAMA, TA and okra molecules. This interaction results in a slight shift of the absorption peak of the hydroxyl stretching vibration mode in HA@TA-Okra to the low frequency direction, accompanied by an increase in peak shape complexity, reflecting the formation of a hydrogen bond network. Furthermore, the FTIR spectra showed a new and more complex absorption characteristic peak in the range of 1700–1750 cm^− 1^, which could be clearly attributed to the vibration of the ester bond (C = O-O-C) formed inside the HA@TA-Okra hydrogel. Compared with the standard peak shape of a single methacrylate group, the absorption peak here shows higher complexity. This is speculated to be due to the formation of various types of ester bond structures inside the hydrogel, including possible spatial conformational differences and different bonding environments, which together contribute to the complex spectral response in this band.

In the in vitro degradation experiments, the degradation behavior of HAMA, HA@TA, HA@Okra, and HA@TA-Okra hydrogels within a PBS environment were systematically evaluated. Specifically, the experimental data unequivocally demonstrated that, by day 5, the degradation rate of all hydrogels attained approximately 40%, providing compelling evidence for the creation of favorable conditions for cell proliferation through partial degradation during the initial stages (Fig. 1H). As the experimental duration progressed, the degradation rate gradually decelerated, plateauing at a relatively stable range of 60-80% after 25 days. This trend underscores the exceptional controllability of HA@TA-Okra hydrogels in terms of biodegradation rates and TA/okra release profiles. We believe that HA@TA-Okra hydrogel its long-term degradation products may mainly include phenolic and quinone degradation products of hyaluronic acid fragments and tannins, as well as small molecules such as sugars and amino acids in okra extract [40–42]. Most of these degradation products are biocompatible and can be excreted from the body in urine, feces, or breath. However, further clinical observations and validation are needed to ensure its long-term safety.

Regarding swelling tests, as depicted in Fig. 1I, all tested hydrogels exhibited marked increases in swelling ratios. Expansion equilibrium was reached within approximately 24 h, with swelling ratios ranging from 310 to 335%. This finding underscores the highly stable cross-linked network structure of these hydrogels, enabling swift and efficient water absorption and retention. Furthermore, existing literature has definitively stated [43] that high swelling rates are associated with a well-developed porous network structure in hydrogels. Although our designed hydrogel has good light-curing and mechanical properties and stability, the long-term stability and storage conditions of the hydrogel need to be further investigated to ensure that the hydrogel maintains its properties unaffected during storage and transportation.

### Biocompatibility, antioxidant potential, and antibacterial efficacy of HA@TA1.25/2.5/5 hydrogels

**Fig. 2:**
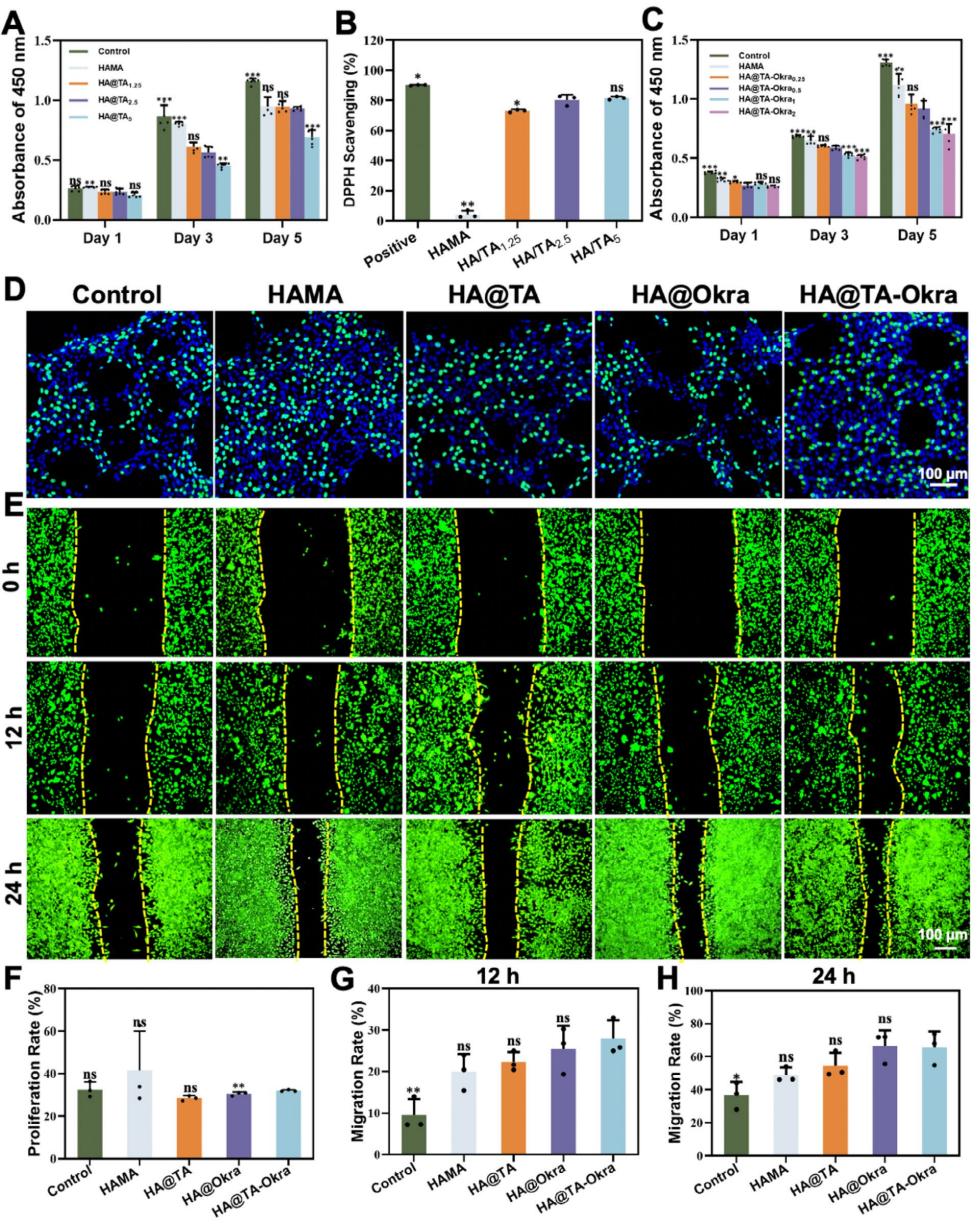
The cytotoxicity and in vitro cell migration behavior of hydrogels. (**A**) Cytotoxicity of L929 cells co-cultured with hydrogels extract medium containing different concentrations of TA for 1,3 and 5 days. (**B**) Determination of DPPH clearance by HAMA and HA@TA_**1.25/2.5/5**_ hydrogels. (**C**) Cytotoxicity of L929 cells co-cultured with hydrogels extract medium for 1,3 and 5 days. (**D**) EdU fluorescence image. (**E**) The migration ability of different hydrogel extracts after co-culture with L929 cells. (**F**) EdU statistical chart. Quantification of margin closure rate between different groups at (**G**) 12 h and (**H**) 24 h (*P < 0.05, **P < 0.01, ***P < 0.001)

A systematic assessment of the biocompatibility characteristics of HA@TA_1.25/2.5/5_ hydrogels were conducted. As depicted in Fig. 2A, all groups exhibited a trend of cellular proliferation over time. At the fifth-day time point, both HA@TA_1.25_ and HA@TA_2.5_ groups maintained a high level of cell proliferation rates comparable to the HAMA group (*P* > 0.05), strongly supporting their superior biocompatibility. Cell proliferation in HA@TA_5_ group was slower than that in HA@TA_1.25_ and HA@TA_2.5_ groups (*P* < 0.001). It may be due to the excessively high TA concentration (5%) surpassing the safe threshold tolerable by cells.

Addressing the prevalent oxidative stress challenge in infected wound environments, the DPPH radical scavenging assay was employed to quantitatively evaluate the in vitro antioxidant efficacy of HA@TA_1.25/2.5/5_ hydrogels. As evident in Fig. 2B, a marked increase in DPPH scavenging rate was observed with the escalation of TA concentration. Specifically, the HA@TA_1.25_ group achieved approximately 73% DPPH scavenging, while both HA@TA_2.5_ and HA@TA_5_ groups exhibited superior scavenging efficiencies exceeding 80%. This finding underscores the potent antioxidant potential of TA’s polyphenolic structure [44–46] and further confirms that modulating TA concentration can effectively enhance the antioxidant properties of the hydrogels. In contrast, the HAMA hydrogel performed poorly in the DPPH scavenging test, with a negligible scavenging rate of 4%.

In exploring the antibacterial properties of HA@TA_1.25/2.5/5_ hydrogels, representative *E. coli* and *S. aureus* were selected as target strains. As shown in Figure S2, although pure HAMA hydrogels lack direct antibacterial activity, their unique three-dimensional network structure acts as a physical barrier to some extent, limiting bacterial growth and proliferation. Upon the introduction of TA at sufficient concentrations, the antibacterial efficacy of the hydrogels was significantly enhanced. The HA@TA_2.5_ hydrogel demonstrated near-complete inhibition (approximately 100%) against both test strains, an effect comparable to that of the higher-concentration HA@TA_5_ group. Integrating cytocompatibility with antibacterial and antioxidant efficacy, the HA@TA_2.5_ (HA@TA for short) was screened to maximize therapeutic effects in practical applications.

### Determination of concentration of okra extract

Among the myriad of biomaterials, okra extract is a promising candidate for the development of skin repair dressings due to its richness in terpenes, phenolics, and flavonoids, which have significant antioxidant properties. In this study, HAMA, HA@TA-Okra_0.25_, HA@TA-Okra_0.5_, HA@TA-Okra_1_, and HA@TA-Okra_2_ were established to identify the optimal hydrogel formulation that best promotes cell growth and functionality. As depicted in Fig. 2C, cell proliferation showed an increasing trend in all groups. However, the results on days 3 and 5 showed an inverse trend in cell proliferation rate with increasing concentration of okra extract. With reference to relevant literatures, HA@TA-Okra_0.5_ hydrogel is selected as the follow-up study.

### The biocompatibility, migration, and antibacterial capacity of HA@TA-okra hydrogels

In order to further examine the properties of HA@TA-Okra_0.5_ hydrogel (HA@TA-Okra for short) and investigate its specific effects on cell behavior, we used EdU cell proliferation assay. As shown in Fig. 2D and F, the results unequivocally demonstrated that L929 cells retained proliferative activity across all tested groups. The HAMA group exhibited the highest cell proliferation rate, attributed to the unique biological function of hyaluronic acid in facilitating extracellular matrix remodeling. The proliferation rates in the HA@TA, HA@Okra and HA@TA-Okra groups were all slightly lower than HAMA group, the differences were statistically insignificant, indicating that the introduction of TA and okra did not adversely affect the proliferative capacity of L929 cells.

Additionally, a scratch assay was conducted to assess the effects of HAMA, HA@TA, HA@Okra, and HA@TA-Okra on cell migration ability. As illustrated in Fig. 2E, G and H, after incubation for 12 h, the healing rates of HAMA, HA@TA, HA@Okra and HA@TA-Okra groups were 19.95%, 22.36%, 25.46% and 27.93%, respectively, while those of the control group were 9.5%. In contrast, the hydrogel group showed efficacy in promoting cell migration. After 24 h, the migration rate of the control group was still lower than that of the other hydrogel groups. The wound healing rates of all hydrogel groups ranged from 50% ~ 65% with no significant difference between groups, further substantiating the immense potential of hydrogel materials in promoting tissue regeneration and wound healing.

**Fig. 3:**
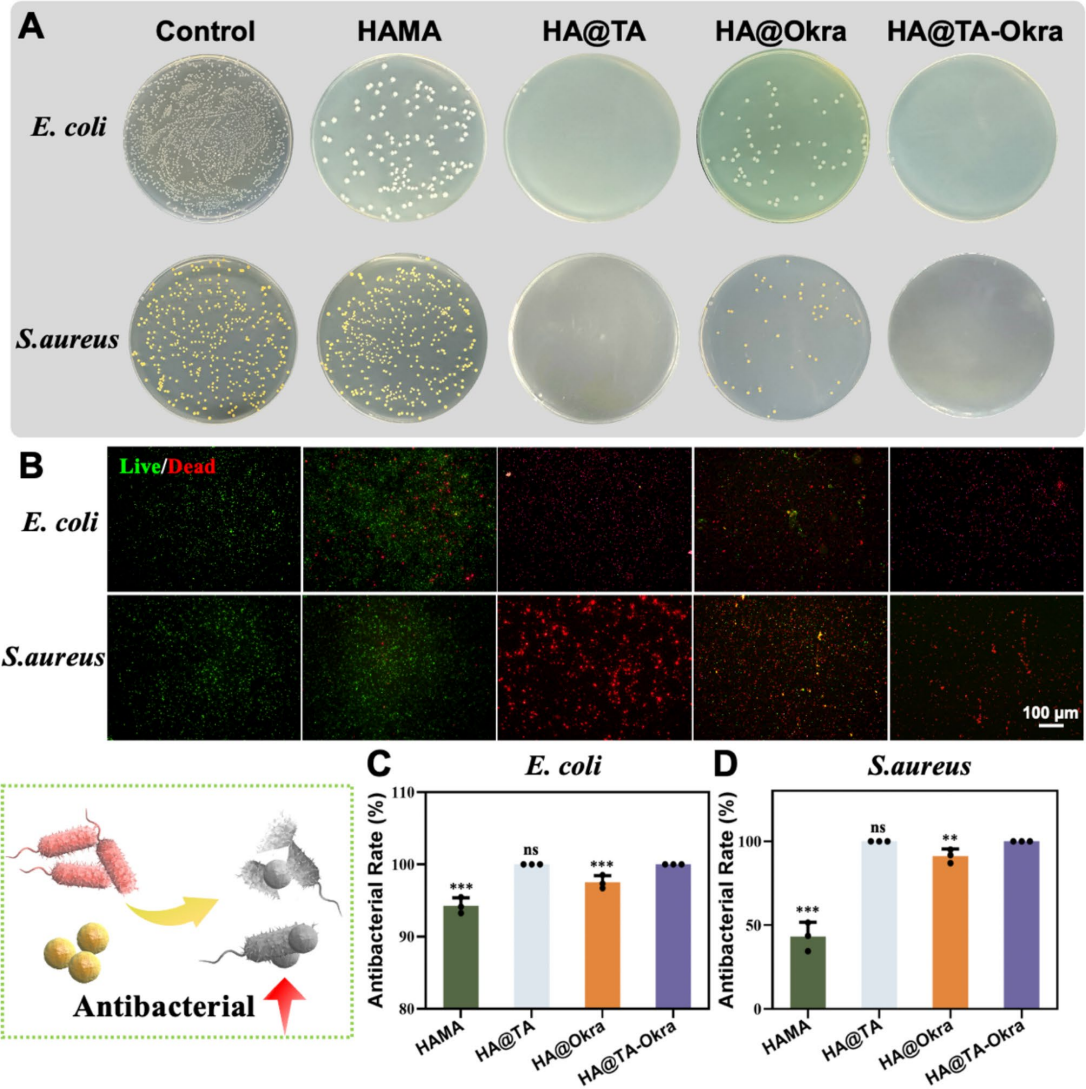
Determination of antibacterial activity of hydrogel against *E. coli* and *S. aureus in vitro*. (**A**) Bacterial plate counting. (**B**) Live/dead staining of *E. coli* and *S. aureus* treated with different hydrogels (green: live bacteria, red: dead bacteria). Statistics of antibacterial rate of different hydrogels against (**C**) *E. coli* and (**D**) *S. aureus*. (**P < 0.01, ***P < 0.001)

The surface antibacterial test results presented in Fig. 3A revealed both the HA@TA and HA@TA-Okra groups exhibited remarkable bactericidal rates exceeding 99% within 4 h. This remarkable performance underscores the potent antibacterial efficacy of hydrogels containing TA. TA is rich in phenolic hydroxyl groups, which contribute to its effective adsorption and accumulation on negatively charged bacterial cell walls, thereby inhibiting bacterial growth and proliferation. This mechanism aligns closely with previous literature findings [47, 48]. To comprehensively evaluate the hydrogels’ antibacterial properties, a live-dead bacterial staining assay was conducted (Fig. 3B). The results indicate that *E. coli* and *S. aureus* in the control group were almost entirely labeled with green fluorescence, indicating significant bacterial activity. In contrast, the HAMA group displayed a weak antibacterial effect, evidenced by sparse red fluorescence, potentially attributed to physical confinement of bacteria by its structure. The HA@Okra group demonstrated a more pronounced antibacterial effect, with a significant reduction in green fluorescence and an increase in red fluorescence. The HA@TA and HA@TA-Okra groups almost exclusively exhibited red fluorescence, a strong indication of their effective bactericidal action. The antibacterial rate statistics in Fig. 3C and D further confirmed our observations. Compared with HAMA group and HA@Okra group, HA@TA-Okra has a significant antibacterial rate (*P* < 0.001), providing quantitative support for the antibacterial efficacy of the HA@TA-Okra hydrogels.

### Investigation into the antioxidant properties and detection of inflammatory cytokine secretion

**Fig. 4:**
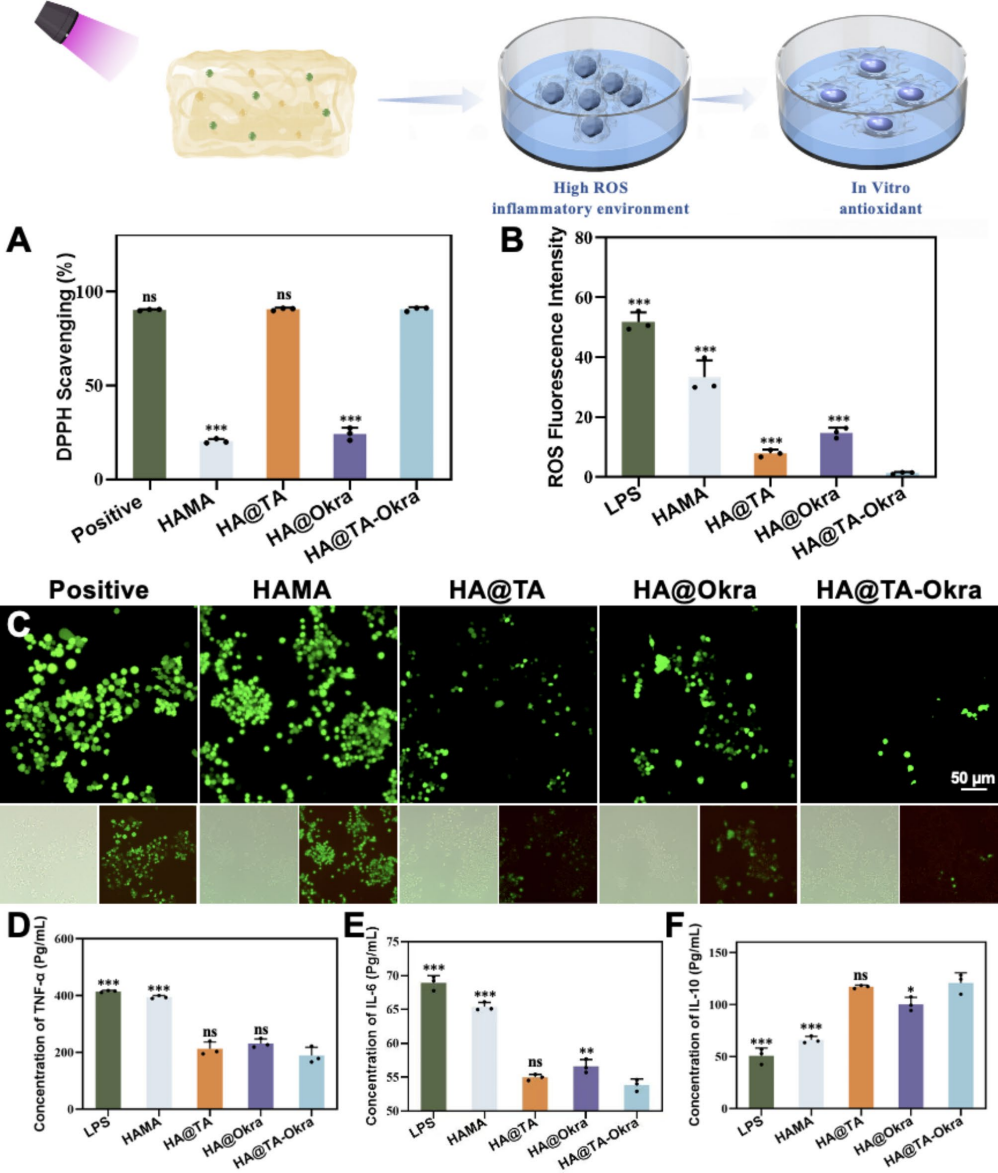
Antioxidant and anti-inflammatory activities of hydrogels in vitro. (**A**) Determination of DPPH clearance by different hydrogels. (**B**) Represents ROS fluorescence intensity. (**C**) Intracellular ROS detection under different hydrogels (Green fluorescence indicates ROS). The secretion of (**D**) TNF-α and (**E**) IL-6 (**F**) IL-10 by RAW264.7 cells on different samples were quantitatively analyzed. (*P < 0.05, **P < 0.01, ***P < 0.001)

In the inflammatory response triggered by skin injury, the accumulation of immune cells at the injury site is often accompanied by an excessive release of ROS. The moderate levels of ROS play a pivotal role in the body’s defense mechanisms, facilitating the initial healing process of wounds [49, 50]. However, its overaccumulation leads to the formation of oxidative stress, subsequently damaging cells and impeding the smooth transition of wounds from the inflammatory to the proliferative phase, ultimately affecting the progress healing [51]. To evaluate the antioxidant capability of HA@TA-Okra hydrogels, this study designed and conducted experiments involving DPPH radical scavenging assays. As depicted in Fig. 4A, the HA@Okra group exhibited approximately a 25% enhancement in DPPH radical scavenging rate compared to the HAMA group, indicating that the incorporation of okra effectively bolstered the antioxidant baseline performance of the hydrogel. However, the antioxidant properties of HA@Okra group were significantly lower than those of HA@TA-Okra group (*P* < 0.001), indicating that TA was the main antioxidant in HA@TA-Okra hydrogel. Notably, upon the integration of TA into the system, both the HA@TA and HA@TA-Okra groups witnessed a substantial leap in scavenging rates, surpassing 90% (no significant difference between the two groups). This remarkable performance can be attributed to the abundant ortho-phenolic hydroxyl groups in TA molecules, which act as efficient hydrogen donors, promptly responding to and neutralizing various types of ROS, including DPPH radicals, hydroxyl radicals, and superoxide anions [52].

To more intuitively reflect the ROS scavenging efficacy of the hydrogels at the cellular level, this study employed DCFH-DA fluorescent probe technology to quantitatively monitor ROS levels within RAW264.7 cells. The DCFH-DA probe permeates cell membranes, gets oxidized by ROS within the cell, and produces DCF, which emits intense green fluorescence, enabling visual assessment of intracellular ROS levels. As shown in Fig. 4B and C, the HA@TA-Okra group (1.29 ± 0.31) exhibited significantly reduced green fluorescence intensity compared to the positive control group, which indicating its potent antioxidant activity in the intracellular environment. In contrast, the cells of HAMA group (33.37 ± 5.56) displayed higher green fluorescence intensity, indicating the production of substantial ROS under LPS stimulation, which the HAMA hydrogel failed to significantly inhibit or scavenge. It is worth noting that ROS levels in the HA@Okra group (14.73 ± 1.69) and the HA@TA group (7.85 ± 1.17) decreased moderately but were higher than those in the HA@TA-Okra group and lower than those in the HAMA group, indicating that both okra extract and TA could enhance the antioxidant activity of HAMA hydrogel.

Macrophages, a pivotal component of the innate immune system, play a crucial role in regulating tissue inflammation through their dynamic polarization states. Specifically, M1 macrophages predominantly execute proinflammatory functions, whereas M2 macrophages are inclined to secrete anti-inflammatory factors to facilitate tissue repair and regeneration [53–56]. The immunomodulatory properties of HA@TA-Okra hydrogel on macrophages in vitro were investigated through a series of experiments. RAW 264.7 cells were utilized as a model to simulate their interaction with hydrogel extracts in vivo by co-incubating. LPS was employed as an inflammatory inducer to stimulate macrophage polarization toward the M1 phenotype and as a positive control. The experimental outcomes (Fig. 4D-F) revealed that the experimental groups containing TA and okra components exhibited significant advantages in regulating inflammatory mediator expression compared to the LPS and HAMA groups (*P* < 0.001). Specifically, these experimental groups demonstrated a marked decrease in the expression levels of proinflammatory cytokines (such as TNF-α and IL-6) from M1 macrophages, accompanied by a substantial increase in the secretion of anti-inflammatory cytokines (such as IL-10) from M2 macrophages. Among them, the HA@TA-Okra hydrogel group was particularly prominent in its anti-inflammatory effects. This phenomenon may be attributed to the synergistic enhancement of the anti-inflammatory mechanisms of TA and okra components [56, 57]. The precise anti-inflammatory mechanisms of okra remain to be fully elucidated, preliminary evidence indicates the potential application of its extracts in the field of anti-inflammation [41].

### In vitro blood compatibility of HA@TA-okra hydrogels

**Fig. 5:**
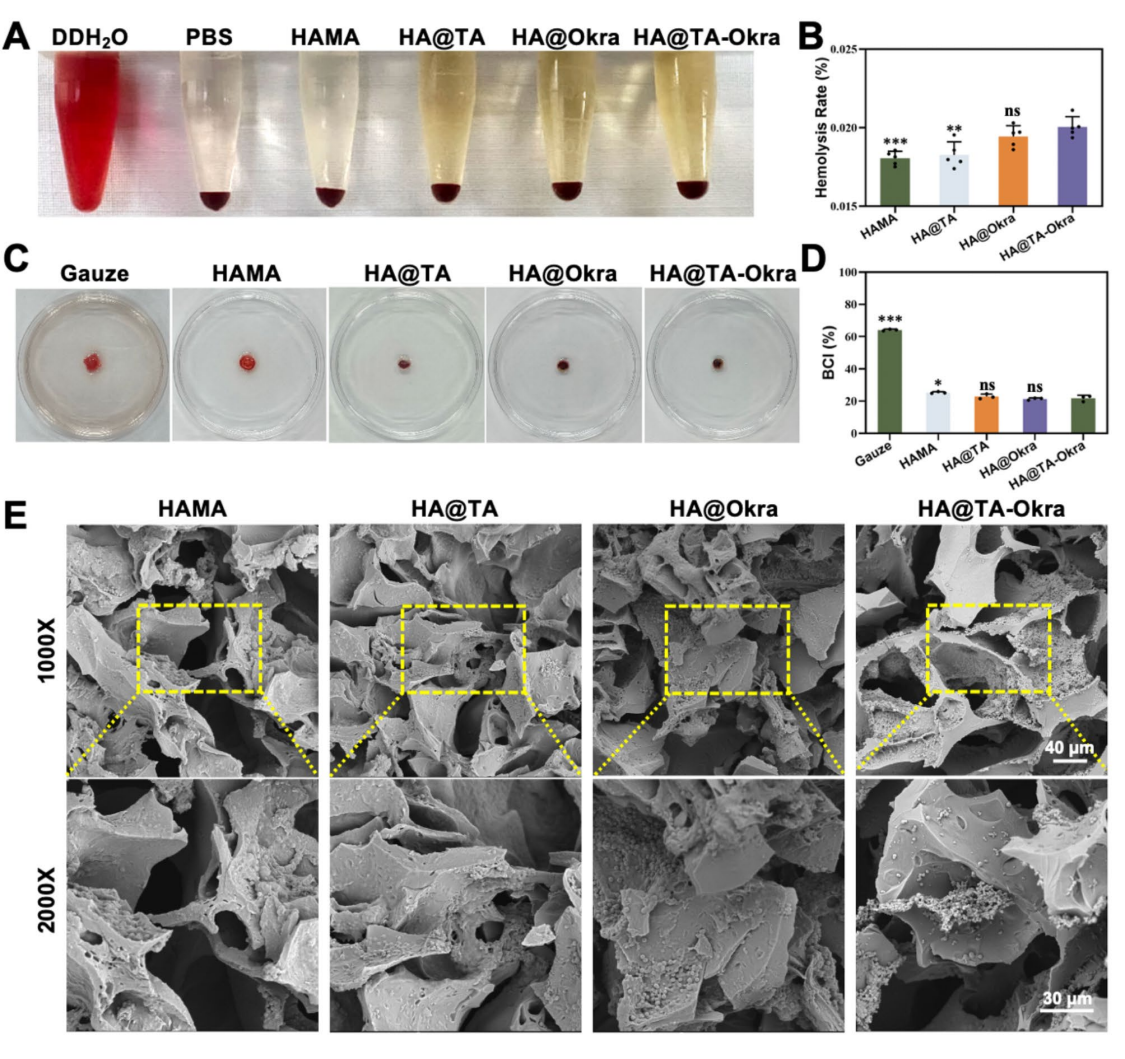
Blood biocompatibility and coagulation properties. (**A**) Hemolysis test photographs of different hydrogels. (**B**) Hemolysis rate. (**C**) Blood absorption photos after gauze and different hydrogels contacted blood and soaked in PBS for 1 min. (**D**) BCI statistical results. (**E**) SEM images of blood cell and platelet adhesion on different hydrogels. (*P < 0.05, **P < 0.01, ***P < 0.001)

The blood compatibility of HAMA, HA@TA, HA@Okra and HA@TA-Okra hydrogels were methodically assessed in this study. Deionized water and PBS were served as positive and negative controls, respectively. As depicted in Fig. 5A and B, the hemolysis rates of all hydrogel groups were markedly below the 5% threshold stipulated by the ASTM F756-2008 standard. This outcome underscores the exceptional safety and compatibility of all hydrogels within a blood environment.

BCI is a key coagulation evaluation index, the lower the BCI value, the better the coagulation effect. As shown in Fig. 5C and D, compared to the gauze groups, all hydrogel groups exhibited significantly reduced BCI values, indicative of their superior coagulation abilities. The HA@Okra and HA@TA-Okra hydrogel groups demonstrated even lower BCI values (21.38% and 21.79%, respectively). Compared with HAMA group, HA@Okra and HA@TA-Okra groups had better coagulation effect (*P* < 0.05). This suggested that the incorporation of okra components may enhance the coagulation properties of the hydrogels by activating platelets, promoting platelet adhesion and coagulation factor release [32]. Furthermore, erythrocyte aggregation at wound surfaces is critical in the formation of an effective hemostatic barrier [58]. The SEM images (Fig. 5E) confirmed that erythrocytes and platelets adhered well and maintained a normal morphology on the surface of the hydrogels, which further showed their good blood compatibility.

### In vivo hemostatic properties

**Fig. 6:**
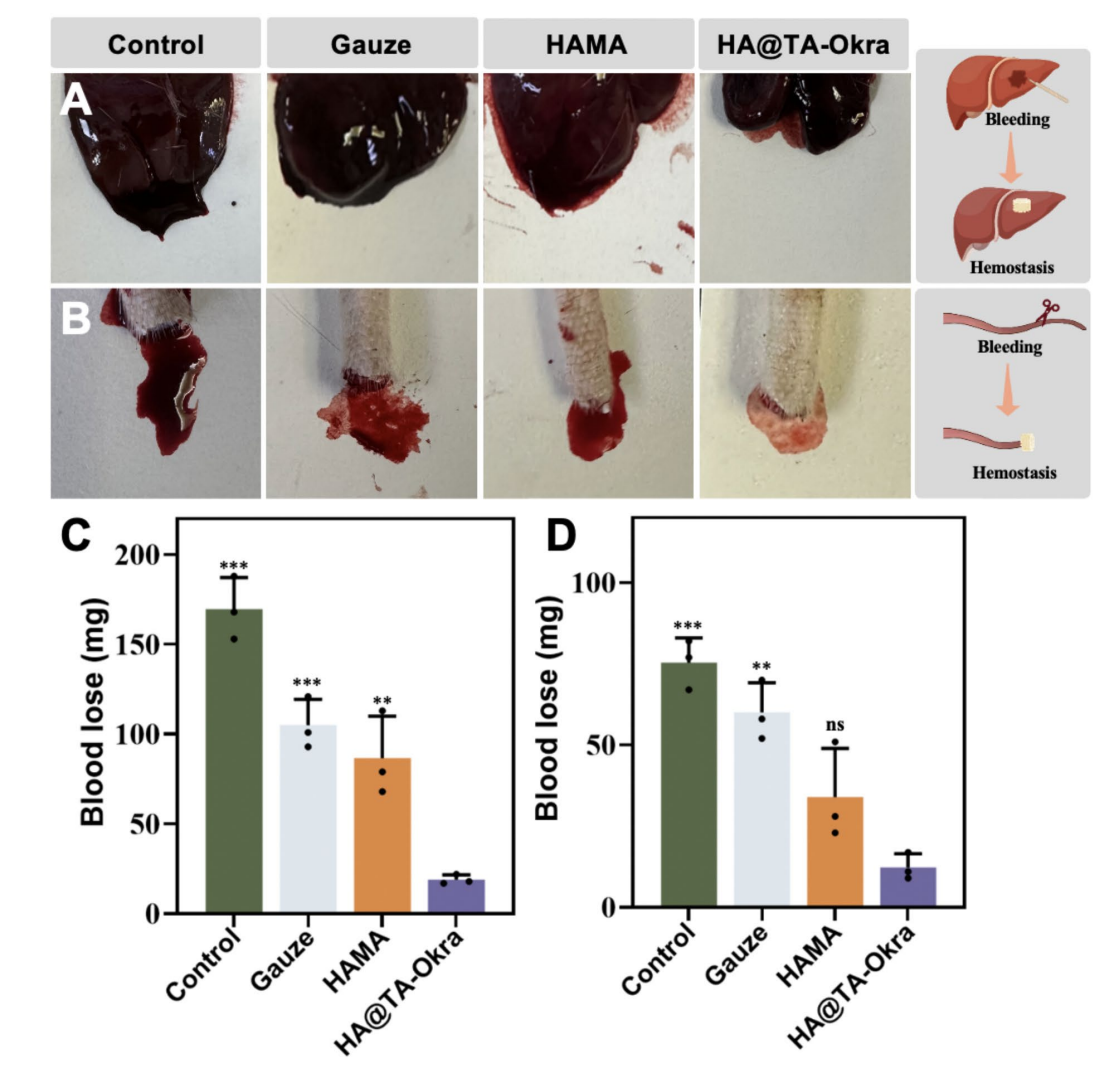
The hemostatic properties of different hydrogels in vivo. Photos of different hydrogel hemostatic properties using (**A**) liver incision and (**B**) tail amputation models. Blood loss of different hydrogels in the (**C**) liver incision and (**D**) tail amputation models. (** *P* < 0.01, *** *P* < 0.001)

Through the above series of in vitro experiments, we chose the HA@TA-Okra hydrogel with relatively best performance for the next in vivo experiments. To further substantiate the hemostatic efficacy of the HA@TA-Okra hydrogels in authentic physiological settings, hemostasis experiments were designed and conducted on two bleeding models: rat liver incision and tail amputation. As illustrated in Fig. 6A and C, in the liver incision model, blood loss was significantly lower in the HA@TA-Okra hydrogel group (19 ± 5 mg) than in the other groups, followed by the HAMA group (86 ± 23 mg). This finding highlights the robust blood absorption capacity of HAMA substrates due to their three-dimensional porous structure. Okra components may confer a remarkable enhancement of HAMA coagulation properties by increasing coagulation factor activity and optimizing platelet function [32]. Based on Fig. 6B and D, it can be found that in the tail amputation model, the amount of bleeding in the HAMA group was higher than that in the HA @ TA-Okra group. This may be due to that although the HAMA group has a stable three-dimensional porous structure, the porous three-dimensional structure is saturated when the amount of bleeding is large, and the residual blood volume cannot be excessively absorbed. After loading okra, it can enhance platelet adhesion, cause platelets to form thrombus to block the wound, and initially achieve hemostasis.

### Wound healing in a full-thickness skin defect model in rats

**Fig. 7:**
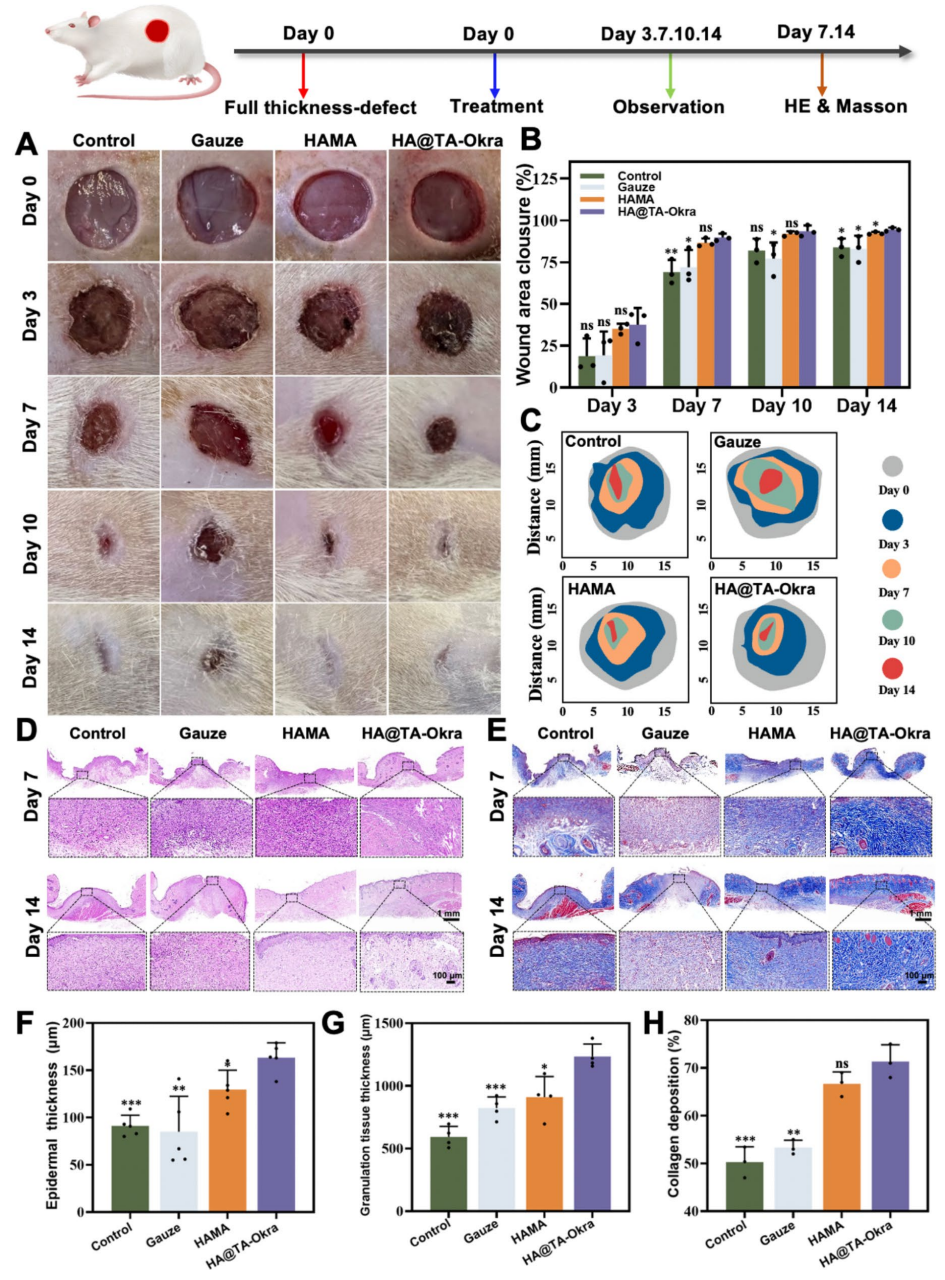
HA@TA-Okra promotes full-thickness skin defect wound healing. (**A**) Representative photos of wounds in different groups after 0,3,7,10 and 14 days of treatment. (**B**) Quantification of wound area in each group. (**C**) The wound treatment pattern of different groups. (**D**) H&E and (**E**) Masson staining images of wound defects on day 7 and day 14 in different groups. (**F**) Skin thickness statistics. (**G**) Granulation tissue thickness statistics. (**H**) Collagen percentage statistics. (*P < 0.05, **P < 0.01, ***P < 0.001)

Investigating the therapeutic potential of HA@TA-Okra hydrogel in tissue repair through a rat model of full-thickness skin defects. As depicted in Fig. 7A and B, the HA@TA-Okra hydrogel group showed a pronounced healing trajectory with a progressive reduction in wound area. During the early stage of healing (day 7), the HA@TA-Okra hydrogel group showed a significant reduction in wound area compared to the other groups. As healing progressed (Day 14), the wounds of the hydrogels in the HAMA and the HA @ TA-Okra group were smaller than those in the control and the gauze group. HAMA and HA @TA-Okra hydrogels can fill the wound, avoid the invasion of external bacterial microorganisms, and accelerate the wound healing rate. In addition, the wound healing effect of HA @ TA-Okra group was statistically different from that of the other three groups (*P* < 0.05), indicating that the hydrogel with TA and okra had a better effect on wound repair. Quantitative analysis of wound healing rates (Fig. 7C) illustrated the remarkable advantages of HA@TA-Okra hydrogel in accelerating wound repair.

To further elucidate the healing mechanism, we collected specimens from each group on day 7 and day 14 and used H&E staining and Masson staining for histological evaluation. The results of H&E staining (Fig. 7D, F and G) showed that the epidermis and granulation tissue layers of HA@TA-Okra group were significantly thicker than those of control and gauze groups on the day 14, indicating that HA@TA-Okra had a positive effect on skin tissue regeneration and remodeling. The main component of hyaluronic acid is the extracellular matrix, which can enhance collagen deposition, epithelial formation and wound angiogenesis. Compared with the HAMA group, the thickness of the epidermis and granulation tissue layer of the HAMA hydrogel loaded with effective anti-inflammatory and hemostatic components was higher, and the healing effect was better (*P* < 0.05). Moreover, Masson staining analysis (Fig. 7E and H) showed that the collagen deposition rate was 71% in the HA@TA-Okra group, which was significantly higher than the 50% in the control group (*P* < 0.001), 53% in the gauze group (*P* < 0.01) and 66% in the HAMA group. This discovery strongly supports the pivotal role of HA@TA-Okra hydrogel in enhancing collagen fiber synthesis and deposition.

### Evaluation of healing efficacy in *S. aureus*-infected wounds

**Fig. 8:**
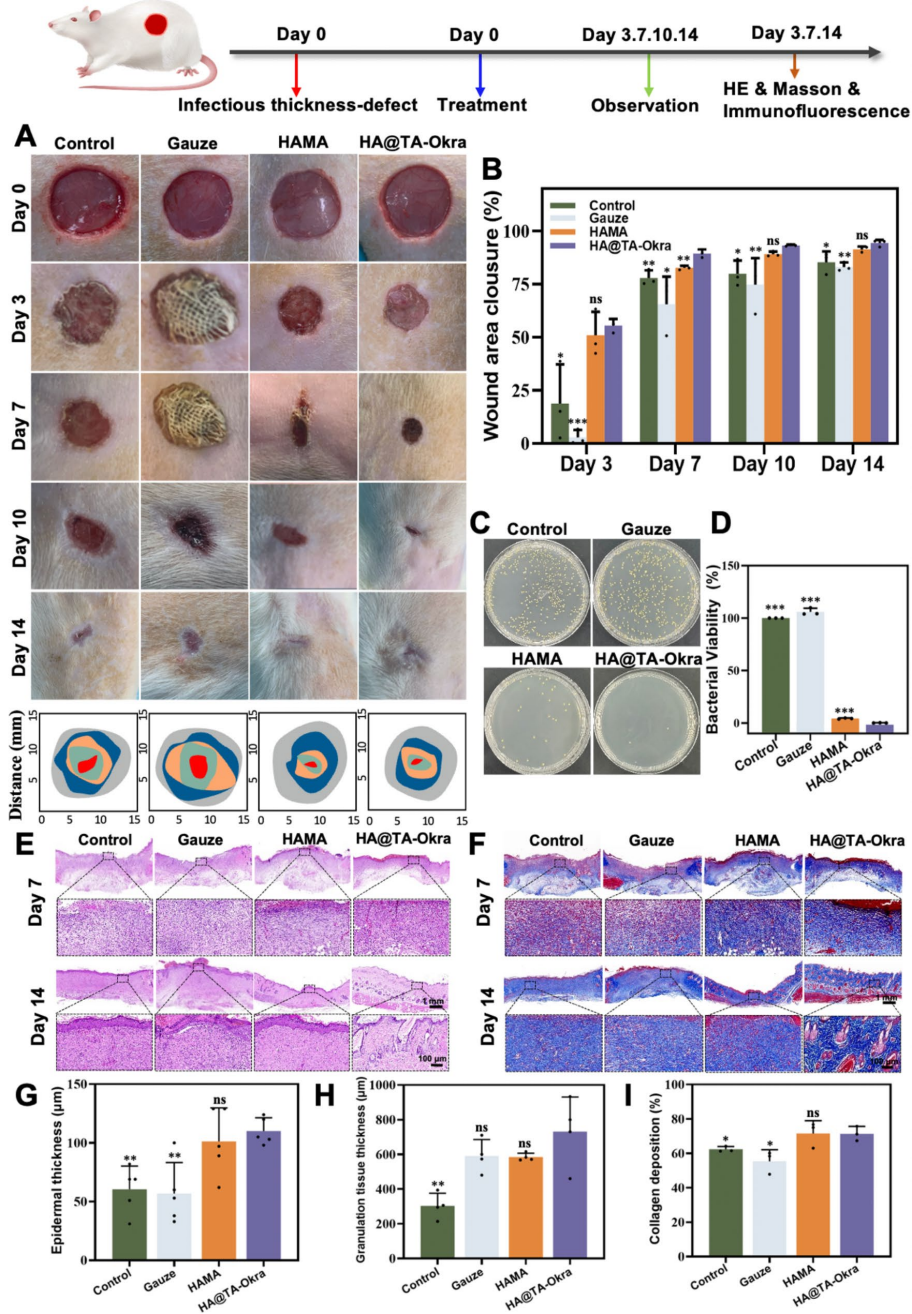
HA@TA-Okra promotes infectious wound healing. (**A**) Infected model wound pictures after 0,3,7,10 and 14 days of treatment. (**B**) Quantification of wound area in each group. (**C**) Photos of infected skin *S. aureus* on the day 3. (**D**) Bacterial colony count statistics. (**E**) H&E and (**F**) Masson staining images of wound defects in different groups on day 7 and day 14. (**G**) Skin thickness statistics. (**H**) Granulation tissue thickness statistics. (**I**) Collagen percentage statistics. (*P < 0.05, **P < 0.01, ***P < 0.001)

This study aimed to systematically assess the healing potential of HA@TA-Okra hydrogel on infected wounds by constructing a full-thickness skin defect model in rats infected with *S. aureus*. As depicted in Fig. 8A and B, both the HAMA and HA@TA-Okra groups significantly accelerated the wound healing process during the continuous therapeutic period, with the HA@TA-Okra groups exhibiting particularly remarkable performance. During the initial phase of treatment (Day 3), the HA@TA-Okra groups achieved a wound closure rate of 56%, far surpassing the 20% observed in the control groups (*P* < 0.05) and the 4% in the gauze group (*P* < 0.001). To delve into the antibacterial effect in more depth, we further analyzed bacterial viability in the wounds on day 3 (Fig. 8C and D). The results indicated a complete inhibition of bacterial viability in the HA@TA-Okra groups, whereas bacterial viability was close to 100% in the control and gauze groups. Bacterial viability also decreased significantly in HAMA group (about 10%). This finding highlights the synergistic antibacterial effect of TA and okra components in the HA@TA-Okra groups. As the treatment progressed, all groups demonstrated an increasing trend in wound healing. The HA@TA-Okra groups continued to exhibit the most pronounced healing effects on days 7 and 10, achieving wound closure rates of 84% and 91%, respectively. By day 14, wounds in this group were almost completely healed (approximately 98%), showcasing its exceptional healing capability. The experimental results showed that the healing rate of HA@TA-Okra hydrogel was accelerated from the day 3, and the healing rate on the day 10 and 14 was significantly higher than that of the control group (*P* < 0.05). The healing rate of gauze group was the lowest from the day 3 to 14. This may be because although gauze, as a traditional wound dressing, can simply protect the wound and block the invasion of bacteria, it is easy to dehydrate the wound and adhere to the wound. When changing the dressing, it is easy to cause mechanical re-injury of the new tissue of the wound, which is easy to cause wound bleeding. Based on the above analysis of the change in wound area with treatment time, the superior wound repair ability of HA@TA-Okra hydrogel was confirmed.

To gain insights into histological changes during wound healing, skin samples were collected on days 3, 7 and 14 post-surgery and subjected to H&E staining and Masson staining analysis. On the day 3, it was found that whether H&E or Masson staining, the epidermis, granulation tissue and collagen production of damaged skin were in the early stage of growth. Among them, the inflammation level of HA@TA-okra groups were lower than that of other groups, which may be related to the good antibacterial and antioxidant properties of its contained ingredients. On day 7, wounds in the control and gauze groups were heavily covered with inflammatory cells, hindering new epidermis formation (*P* < 0.05). Although complete epithelialization was not achieved in the HAMA and HA@TA-Okra groups, the significant reduction in inflammatory cell infiltration favored wound healing. By day 14, all hydrogel-treated wounds exhibited complete epidermal coverage. The HA@TA-Okra group displayed regeneration of skin appendages, such as hair follicles and sebaceous glands, indicative of the best re-epithelialization outcome (Fig. 8E, G and H). Furthermore, we chose collagen deposition as an indicator of wound healing quality. On day 14, Masson staining revealed sparse collagen deposition in the control group and abundant collagen accumulation in the hydrogel-treated group. Moreover, the collagen fibers in the HA@TA-Okra group were more orderly arranged, resembling the structural characteristics of normal skin tissue (Fig. 8F and I). Compared with the HA@TA-Okra group, the collagen in the control and gauze groups was not neatly arranged and the content was less (*P* < 0.05). This finding demonstrates the significant advantage of HA@TA-Okra hydrogel in promoting collagen synthesis.

**Fig. 9:**
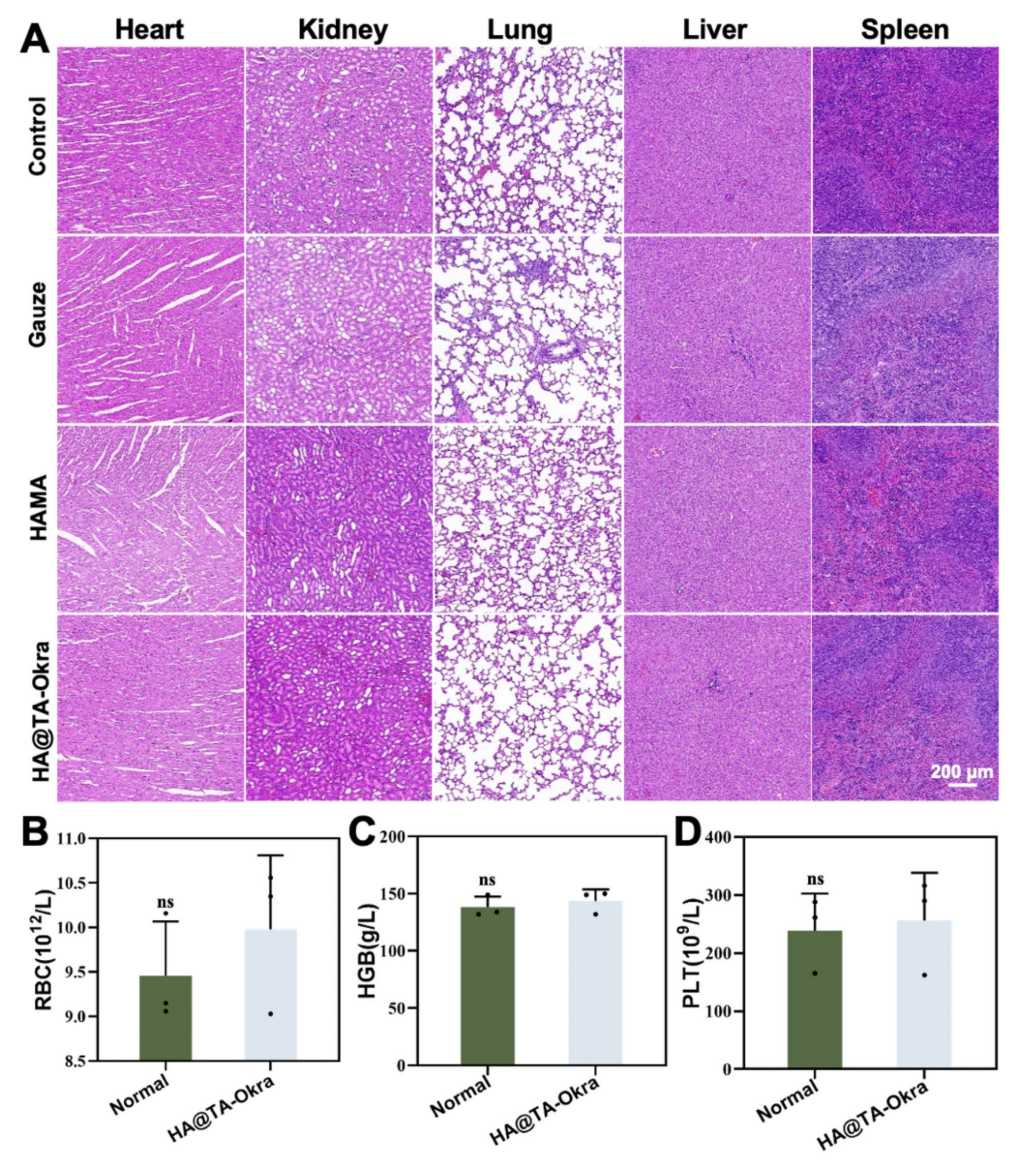
Biocompatibility testing in vivo. (**A**) H&E staining of heart, liver, spleen, lung and kidney. (**B**) RBC (**C**) HGB and (**D**) PLT blood routine test

Finally, the experimental animals were analyzed for histopathology of major organs and routine blood tests to comprehensively evaluate the biosafety of HA@TA-Okra hydrogel. The results indicated that there were no obvious pathological changes in the heart, liver, spleen, lungs and kidneys of the animals in all treatment groups (Fig. 9A-D). Additionally, hematological parameters such as red blood cells, platelets, and hemoglobin remained within normal ranges in the HA@TA-Okra groups, suggesting good biocompatibility and in vivo safety of this hydrogel.

Although hydrogels have shown promising antimicrobial and pro-healing effects in vitro and in animal experiments, it is still a challenge to ensure the suitability of hydrogels for different wound types in actual clinical applications. For example, for complex wounds such as deep burns or chronic ulcers, more personalized treatment protocols and finer hydrogel designs may be required.

### Immunofluorescence analysis

**Fig. 10:**
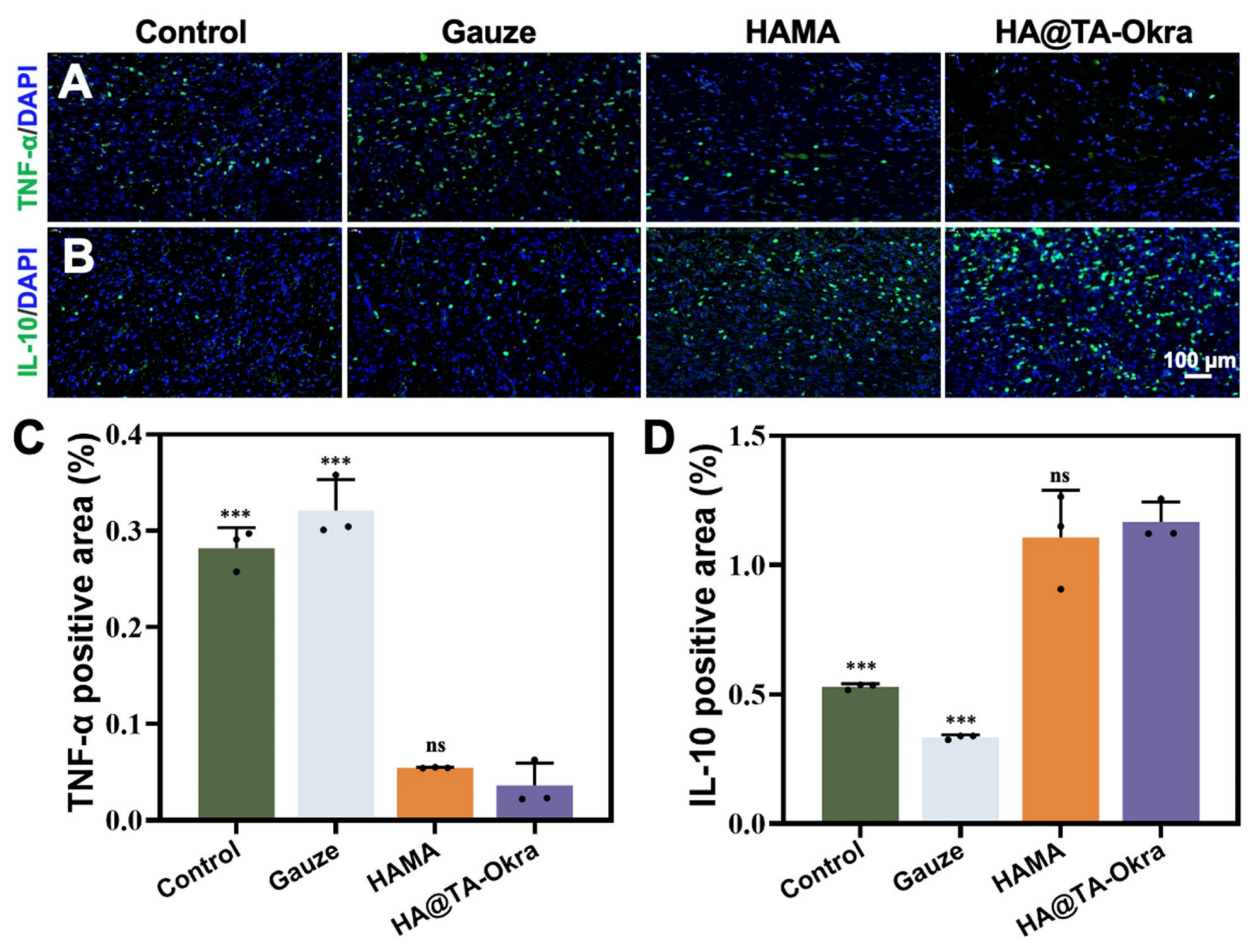
(**A**) Immunofluorescence staining of TNF-α (green) and DAPI (blue) in regenerated skin tissue. (**B**) Immunofluorescence staining of IL-10 (green) and DAPI (blue) in regenerated skin tissue. Immunofluorescence statistical plots of (**C**) TNF - α and (**D**) IL-10. (***P < 0.001)

In this study, immunofluorescence analysis was performed on wound skin at day 14, focusing on elucidating the expression profiles of two pivotal immunomodulatory factors: tumor TNF-α and IL-10. Immunofluorescence images (Fig. 10A and B) showed the expression of TNF-α and IL-10 in different treatment groups. Analysis of the quantitative results (Fig. 10C) demonstrated that the HA@TA-Okra group exhibited the lowest level of TNF-α expression, indicative of its efficacy in mitigating excessive inflammatory responses. In contrast, while the HAMA groups also demonstrated relatively lower TNF-α expression, its effect was slightly inferior to that of the HA@TA-Okra groups. Conversely, the control and gauze groups manifested significantly elevated TNF-α expression (*P* < 0.001), reflecting a high inflammatory state, potentially attributable to the absence of effective immune modulation. On the other hand, the expression level of IL-10 in HA@TA-Okra group (Fig. 10D) was significantly higher than that in control and gauze groups (*P* < 0.001). The increased expression of IL-10 may be closely related to its unique antibacterial properties and enhanced antioxidant capacity.

## Conclusion

In this study, we successfully developed a bioactive skin-mimicking hydrogel wound dressing with multifaceted therapeutic benefits (HA@TA-Okra). Our findings reveal that the incorporation of TA and okra into the HAMA network significantly bolsters the hydrogel’s mechanical properties, ensuring its durability and integrity under local stress conditions. The HA@TA-Okra hydrogels display rapid gelation, favorable swelling behavior, and efficient degradability, coupled with robust antioxidant and antibacterial activities. Furthermore, this hydrogel exhibits exceptional blood compatibility and hemostatic capabilities, outperforming traditional gauze in rat hemorrhage models. In models of full-thickness wounds and Staphylococcus aureus infections, the HA@TA-Okra hydrogels expedited wound repair and regeneration, underscoring its immense potential as an advanced wound dressing material.

In addition to the remarkable effects on HA@TA-Okra hydrogel in promoting infected wound healing, the prospect of its translational application is also worth exploring in depth. First, from the perspective of industrial scalability, the preparation process of HA@TA-Okra hydrogel is relatively simple and key raw materials such as HAMA, TA and okra extract are easily accessible. This opens the possibility of large-scale production and is expected to fulfill the growing clinical demand for efficient wound dressings. Secondly, customized applications of HA@TA-Okra hydrogel also show great potential. As there are various types of wounds, including but not limited to burns, traumas, diabetic foot ulcers, etc., each of which has different needs for dressings, the composition and properties of HA@TA-Okra hydrogel can be flexibly adjusted by adjusting the ratio of raw materials and preparation conditions to meet the treatment needs of different wounds.

In summary, HA@TA-Okra hydrogel not only shows significant advantages in the treatment of infected wounds, but also its broad prospects for translational applications are also worthy of our in-depth research and exploration.

## Electronic supplementary material

Below is the link to the electronic supplementary material.

Supplementary Material 1

## Data Availability

No datasets were generated or analysed during the current study.
