# Supplementary material for: An antibacterial, antioxidant and hemostatic hydrogel accelerates infectious wound healing
Source: J Nanobiotechnology. 2025 Jan 28;23:49. doi: 10.1186/s12951-025-03148-w (PMC11773726; doi:10.1186/s12951-025-03148-w)

**Supporting Information**

**An antibacterial, antioxidant and hemostatic hydrogel accelerates infectious wound healing**

Ziyi Zhou^a, †^, Dengjun Zhang^a, †^, Xuchao Ning ^C, †^, Linbo Jin^a^, Yijing Lin ^a^, Chen Liang^a^, Xin Wen^a^, Tianhao Huang ^a^, Junli Zhou^b,^ *, Yiming Zhang^a,^ *

^a^ Department of Plastic and Cosmetic Surgery, Xinqiao Hospital, Army Medical

University, Chongqing, 400037, China

^b^ Department of Burn and Plastic surgery, The Tenth Affiliated Hospital of Southern

Medical University (Dongguan People's Hospital), China

^C^ Department of Plastic Surgery, Qilu Hospital Qingdao, Cheeloo College of Medicine, Shandong University, Qingdao 266035, China

† Z. Zhou, D. Zhang and X. Ning contributed equally to this work.

*Corresponding authors: 13993185891@163.com (J. Zhou), [zhangyiming@tmmu.edu.cn](mailto:zhangyiming@tmmu.edu.cn) (Y. Zhang).


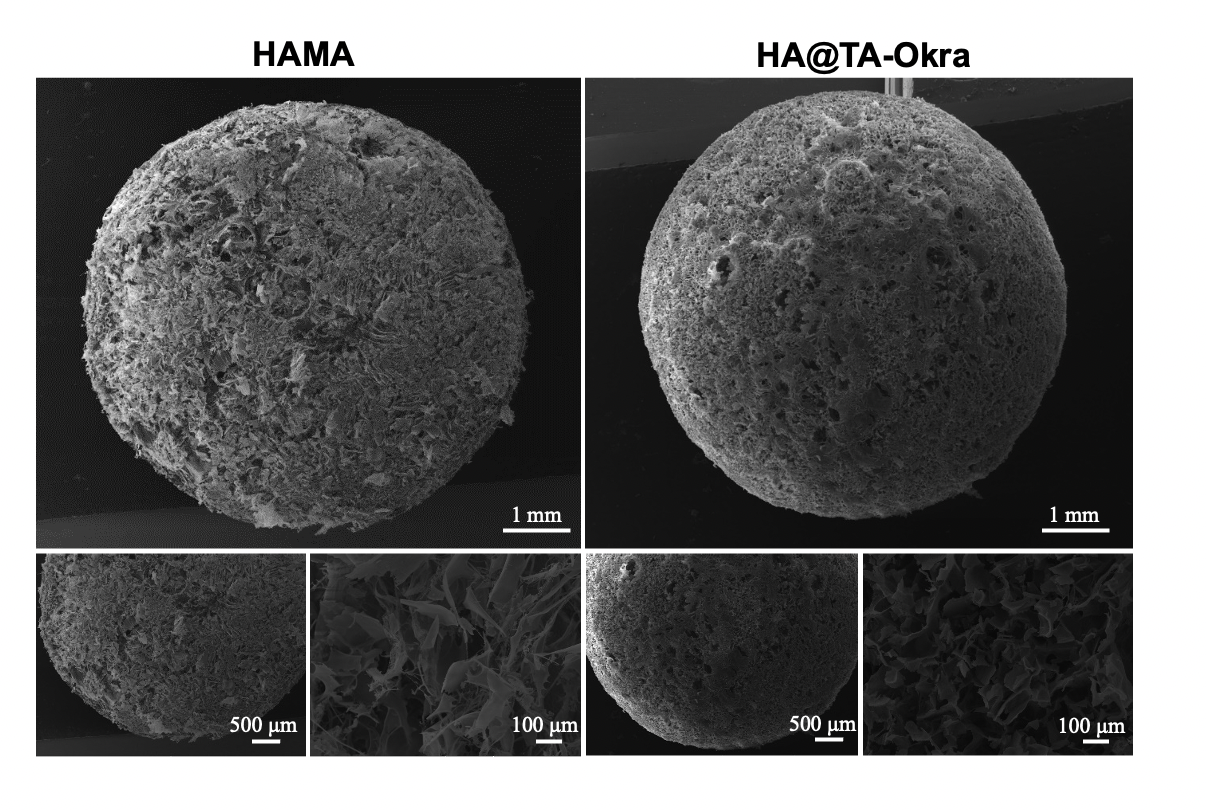


**Fig S1.** Frontal SEM images of HAMA and HA@TA-Okra

**
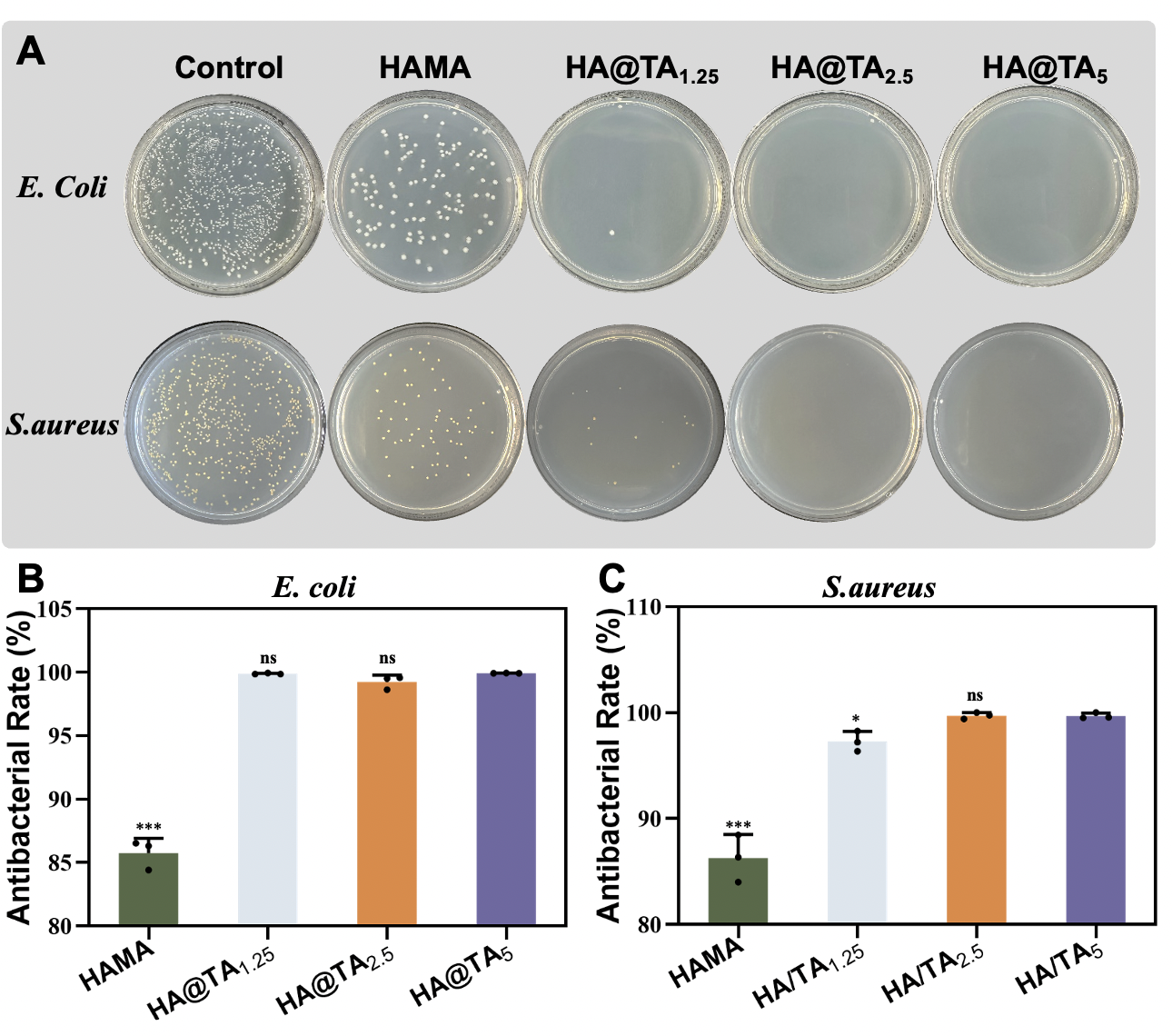
 Fig S2.** Determination of antibacterial activity of HA@TA_1.25/2.5/5_ hydrogel against *E. coli* and *S. aureus*. (A) Bacterial plate counting. Viability of different hydrogels against (B) *E. coli* and (C) *S. aureus*. (****P* < 0.001).


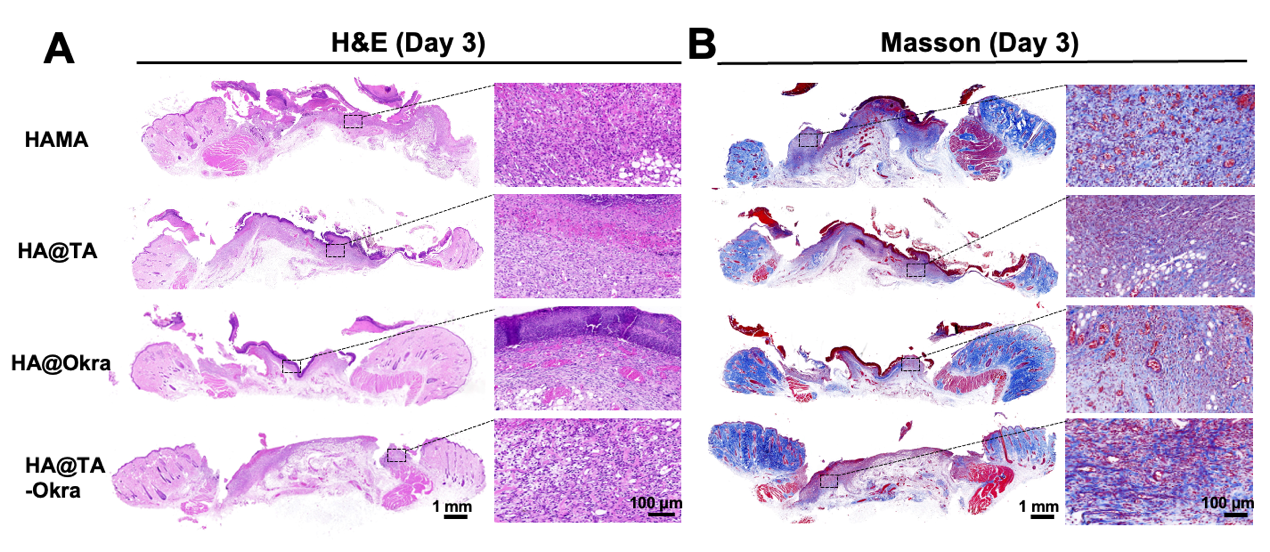


**Fig S3.** (A) H&E and (B) Masson staining images of infected wound defects in different groups on day 3.

**Table 1**


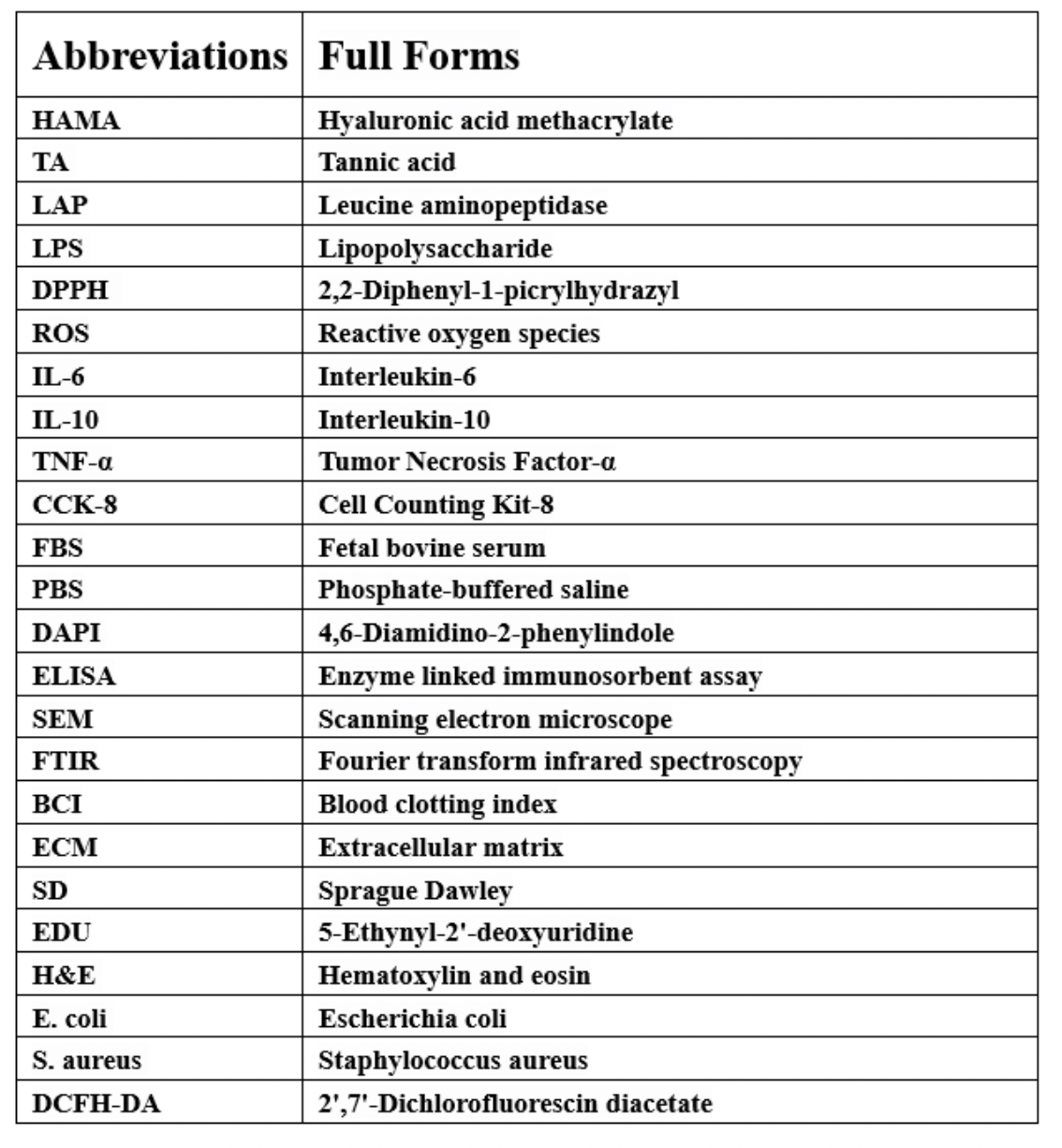

Supplement: Supplementary file 1 — Supplementary Material 1 [file 12951_2025_3148_MOESM1_ESM.docx]
